# Blended teaching of medical ethics during COVID-19: practice and reflection

**DOI:** 10.1186/s12909-022-03431-6

**Published:** 2022-05-11

**Authors:** Min Chen, Lipin Ye, Yucen Weng

**Affiliations:** 1 Institute of Education, Xiamen University, 422 Siming South Road, Siming District, Xiamen, 361005 Fujian China; 2 Research Centre for Medical Humanities, Fujian Medical University, 1 Xuefu North Road, University New Area, Fuzhou, 350122 Fujian China; 3 Health Research Institute, Fujian Medical University, 201B, Administration Building A, 1 Xuefu North Road, University New Area, Fuzhou, 350122 Fujian China

**Keywords:** Medical ethics, Flipped classroom, Blended teaching, COVID-19, Action research

## Abstract

**Background:**

With the advancement of information technology, teachers have become able to overcome the limitations of time and room capacity by carrying out teaching activities online. This practice, however, also presents new challenges. The present study explores how to fully capitalize on the advantages of online and offline teaching and improve the quality and impact of the teaching delivered. This article presents an analysis of the planning, implementation, evaluation, and reflection process of reforming the Fujian Medical University (FJMU) medical ethics course.

**Methods:**

After early attempts using the Small Private Online Course (SPOC) and flipped classroom formats, this paper focuses on the comprehensive active implementation of blended teaching practice. In terms of teaching practice, this research makes targeted improvements to overcome the known shortcomings of SPOCs and flipped classrooms, including the significant preparatory workload and lacking enthusiasm for classroom participation, by redesigning the course and evaluation method and changing the role of the teacher in blended teaching. Subsequently, the study used a stratified sampling method to select 20 students enrolled in the clinical medicine course at Fujian Medical University (FJMU). Their course experience was investigated using a semi-structured interview. Interview content related to evaluating teaching effect was extracted and encoded for subsequent qualitative analysis.

**Results:**

A qualitative analysis of the student evaluation of blended teaching as implemented on the medical ethics course showed that the main factors influencing student engagement are the method of assigning tasks and that of testing learning outcomes. Student participation in class is influenced by the richness of the curriculum resources available and the role played by the teacher.

**Conclusion:**

This research presents a discussion of blended teaching and suggests improvements that can be made to address low student engagement and poor classroom participation. This round of blended teaching was shown to improve learning autonomy and classroom participation and to support students in the development of their clinical abilities and higher-order thinking skills. These findings provide a reference for the implementation of online teaching during the COVID-19 pandemic.

**Supplementary Information:**

The online version contains supplementary material available at 10.1186/s12909-022-03431-6.

## Background

### Motivation of teaching reform

Traditionally, teaching activities are undertaken as classroom lectures [1]. The rapid advancement of information technology, especially the increased speed and availability of internet technology, has facilitated increased means for educators to modify the teaching methods used, achieve teaching objectives, and improve teaching standards [2]. Various online teaching techniques have been used to support learning across different subjects [3]. The MOOC (Massive Open Online Course) format allows thousands of people to access a wide variety of course resources on a publicly available web platform anywhere and at any time; the SPOC (Small Private Online Course) enables teachers to increase interaction with students using platform-based resources within appropriately sized classes; micro-courses concisely present classified educational content in the form of short videos; the flipped classroom transfers the learning initiative to the students so that teachers can deliver in-class content with more depth and efficiency, and blended teaching attempts to integrate traditional teaching with online learning. Reforming teaching methods based on “Internet plus teaching” brings new challenges to teachers and requires new ways of thinking.

To meet the demands of cultivating medical practitioners with higher levels of qualification and decision-making capabilities in the field of ethics, while needing to rely on modern teaching methods, the author (hereafter referred to as the teacher) conducted a reform of a Medical Ethics course at Fujian Medical University over a period since 2015 to incorporate online teaching. Before presenting a comprehensive analysis of the implementation of blended teaching, this study also reports the process of reforming teaching practice using the interim methods of the SPOC and the flipped classroom.

### Initial attempts and problems found

#### SPOC with direct recording

The use of a SPOC to teach Medical Ethics from 2015 used learning resources in the form of 40-minute videos and supporting exercises created in a recording studio. These were uploaded to the online course platform for students to access. The course contained 27 class hours, comprising three chapters and replacing classroom teaching with online recorded lectures, that is, 18 hours of traditional teaching, and 9 hours of watching pre-recorded SPOC videos.

After one semester, it was found that the SPOC provided some advantages over traditional teaching. Firstly, to a certain extent, the online self-study format supported students in developing self-discipline [4]. Secondly, the format overcame the limitations of time constraints and room capacities; students could learn online anytime and anywhere, and any student absent due to illness was allowed to catch up [5]. A comparison conducted using the MOOC format showed that there was no significant difference in academic performance between students taught using the MOOC format and those who attended traditional in-class lectures [6]. The online teaching format greatly improves the flexibility of teaching while not negatively affecting the quality of the teaching delivered. Online teaching may additionally help to improve equality in education [7]. With the only pre-requisites being a network and a computer, high-quality education resources can be made accessible to medical students and medical practitioners in remote areas.

There were, however, problems identified in the early implementation of SPOC teaching. The quality of the PowerPoint slides and videos used in pre-recorded lectures was often poor, with the audio and video streams sometimes becoming de-synchronized, negatively impacting students’ concentration [8]. The course platform only recorded student interactions with fixed activities, such as homework and questions, and failed to capture students’ cognitive learning characteristics or make a quantitative assessment of students’ understanding or performance [9], consequently making it difficult for teachers to observe and evaluate the effectiveness of students’ learning in real-time [10]. The presentation of recordings was monotonous and lacked targeted guidance [11]. Single topics or knowledge units had to be segmented across multiple separate videos, affecting the flow and continuity of the teaching. Finally, there was no guarantee that students would actually watch the videos as required, risking those students with poor self-discipline falling behind [12].

#### Flipped classroom

To overcome the shortcomings of pre-recorded SPOC videos, the teacher introduced the flipped classroom format based on micro-videos to be watched before working through the new material in class. The flipped classroom method was implemented in the teaching of undergraduate-level medical ethics across different years between 2018 and 2020. The format comprised of a resource pool with between 5 and 15 minutes of micro-videos pre-recorded by the teacher. Students completed the learning of declarative knowledge independently by watching micro-videos on the online course platform, discussing the content with classmates online [13], constructing a mind map, and consulting literature resources for learning content [14]. Quizzes were inserted into online videos to test students’ understanding. In the management module of the online platform, teachers could obtain the learning status of each student, including their progress, time spent watching videos and discussing them online, frequency of platform visits, and other completion metrics. This encouraged students to keep up with their learning. In face-to-face classes, teachers used cases to guide students through putting their newly-learned ethical theory into practice, giving them a deeper understanding of how to solve practical problems [15], realizing the internalization of their knowledge into practical ability, and promoting the development of higher-order thinking skills [16]. At the same time, the flexibility of the self-paced learning component of the flipped classroom design addressed the needs of individual students [17], highlighting the autonomy of students in the learning process [18].

Nevertheless, the deficiencies found in the flipped classroom teaching format were that it still relied on students to complete a significant amount of preparation work before class. Some students forgot, got confused, or failed to complete the study in time [19], in turn affecting their subsequent participation in case discussions in the face-to-face sessions. Further, the learning process was predominantly led by the teacher, as the online system did not allow for screening or filtering of comments, students were not fully encouraged to express their personal opinions, and sometimes made meaningless comments without thinking. These aspects of participation were highlighted as requiring improvement [20].

### Research question and research objective

To address these problems, further change and improvement in the teaching method were necessary. Using the medical ethics course at Fujian Medical University as an experimental case, this study explores the available options for using online teaching platforms and aids, aiming to optimize students’ online experiences in undertaking disciplined and self-motivated study in combination with offline teaching, as well as to maximize the combined utility of the two formats.

In the second half of 2020, a redesign of the medical ethics course was carried out. Through sequential planning, implementation, evaluation, and reflection, this redesign aimed to make continuous improvements to the teaching methods and quality of active research. More importantly, based on the previous experience of using the SPOC and flipped classroom formats, this stage of the study aimed to find a more effective and generalizable method for the delivery of medical ethics teaching.

Following the outbreak of COVID-19, canceling offline teaching activities in universities became necessary to prevent the spread of the virus [21]. Throughout the pandemic, the design of the medical ethics course changed further within the format of the flipped classroom. In half a year, through implementing blended teaching across the using “micro-classes”, together with online courses, a complete teaching cycle was delivered and evaluated for second-year clinical medical undergraduates. The teaching content included 9 chapters representing 27 class hour units, totaling 1.5 credits.

## Methods

### Course design

Both flipped classroom and blended teaching formats can be categorized as “learning before discussing”, in that students need to obtain a basic theoretical knowledge of a topic independently through the online course platform before attending an in-person class [22]. Students find the burden of self-study too great and report that it is accompanied by insufficient clear direction [23]. Some students even doubt whether it is worth spending so much effort learning medical ethics rather than learning actual medicine. Blended teaching, therefore, must improve on this to generate enthusiasm for learning medical ethics by redesigning the self-study tasks and teaching.

The objectives of the medical ethics course are giving the students a mastery of the basic knowledge of medical ethics, an understanding of the theoretical basis of medical ethics and the guiding value of its principles and norms in medical practice, and a capability to analyze the moral judgment of medical ethical conflicts. As such, the blended teaching is designed to combine the use of pre-recorded video for independent study, case-based teaching in the online course, and group discussion of cases. Students acquired the basic theoretical knowledge through videos and other teaching materials on the online platform before each online class. Based on self-study in the first instance, the online meeting embraced the teaching resources of the online platform to carry out activities such as explaining key and difficult concepts, analyzing cases, facilitating group discussion, and simulating scenarios. Following the meeting, homework, such as quizzes, was used to test students’ learning outcomes. To avoid students forgetting this new knowledge and to minimize the impact of the fragmentary nature of course content, the course was scheduled as 9 hours of online self-motivated study, 15 hours of online class meetings, and 3 hours of group case discussion and reporting. The timing and content of the online class meetings and pre-recorded videos for students to watch in their own time were structured in a way closely linked to the cohesive aims of the course. Table 1 shows part of the teaching schedule.

**Table 1:** Part of the teaching schedule for the blended delivery of the medical ethics course

**Week**	**Date**	**Class**	**Hours**	**Teaching Content**	**Teaching Space**
3	2020-09-18	1-1	1	Ethics of doctor-patient relationship (learn the basic theoretical knowledge, watch videos 3.1 to 3.4 of Medical Ethics on Chaoxingerya Platform)	Online Self-study
3	2020-09-18	2-3	2	Ethics of the doctor-patient relationship (online course, ability development, case discussion; the crucial point is to analyze the rights and obligations of doctors and patients in the practice of diagnosis and treatment, and discuss causes of doctor-patient conflicts and possible adjustment methods)	Online Meeting Class
4	2020-09-25	1-1	1	Ethics of clinical diagnosis and treatment (learn the basic theoretical knowledge, watch videos 4.1 to 4.7 of Medical Ethics on Chaoxingerya Platform)	Online Self-study
4	2020-09-25	2-3	2	Ethics of clinical diagnosis and treatment (online course, ability development, case discussion; the crucial point is to guide students to analyze the ethical principles in clinical diagnosis and treatment in combination with the situation, and to understand the ethical requirements of diagnosis and treatment at different stages)	Online Meeting Class
6	2020-10-09	1-1	1	Ethics of hospice care and death (learn the basic theoretical knowledge, watch videos 5.1 to 5.3 of Medical Ethics on Chaoxingerya Platform)	Online Self-study
6	2020-10-09	2-3	2	Ethics of hospice care and death (online course, ability development, case discussion; the crucial point is to describe clinical situations to help students understand and master the application of ethics of hospice care and death)	Online Meeting Class

### Qualitative evaluation of the course

In the process of reforming teaching methods to improve teaching quality, it is important to meet the long-term teaching objectives, capture and summarize student experiences, identify problems as they arise, and make the necessary improvements [24]. To evaluate the success of the blended teaching method, indicators of teaching outcome should be observable [25], representative [26], comparable across attributes [27], and objective in reflecting students’ mastery of the topics [28]. Students’ final course grades meet the above criteria, but it should be noted that the marking strategy must be comprehensive. According to the principle of maximizing resources, students’ final marks were composed of 60% final examination and 40% continuous assessment (which itself is comprised 50% course platform learning score, 40% group case report score, and 10% in-class participation and attendance), reflecting the weightings of the level of knowledge mastery, course engagement, and attendance, respectively.

To address the research objective, this study used a qualitative methodology with a constructivist basis and collected data using semi-structured interviews. Over a semester of teaching practice, to ensure a sample reflective of typical course students, student sampling was stratified according to their final course grades. Of the total course intake of 60 students, a fixed percentage of students achieving each grade (A through D) was randomly selected, such that the sample comprised 20 research participants. Semi-structured interviews were conducted, covering teaching content, teaching methods, and teaching objectives. Interviews were transcribed into text for content mining and analysis. An in-depth content mining analysis was conducted to provide statistical analyses of word frequencies and on the semantic distinctiveness of repeated expressions. A qualitative analysis was conducted to determine the main issues with internal relevance to the research topic and to summarize and generalize across experiences.

### Procedure and data analysis

The semi-structured interview outline used in this study (see Appendix) contained 8 questions, including “The teaching resources of the Medical Ethics course include diverse digital resources such as short videos of knowledge points, case studies, test practice questions, discussion topics, etc. What do you think are the features of these teaching resources? Can you give us an example?”, and “The assessment method used in the course this year uses a combination of formative and summative assessment, do you think it accurately assesses student ability? Is this reasonable?”. Each interview lasted approximately 20-30 minutes. After transcribing the audio recordings into text, the transcript of the interviews totaled 23,225 words. Transcripts were coded using NVivo11.0 qualitative analysis software and word frequencies were counted using ROST CM6 to obtain the most significant influential curriculum elements representing students’ dedication and engagement, in-class initiative, and clinical decision-making capability. 93 open codes, 6 axial codes, and 3 selective codes were extracted respectively (see Table 2).

**Table 2:** Semi-structured interview data after coding and word frequency analysis

**Open Coding** (**Partial)**	**Axial Coding**	**Selective Coding**	**Material Sources**	**Reference Nodes**
“The teacher starts the class by explaining what needs to be mastered and what just needs to be understood.” “If there is a difficult area, the teacher will talk more during the class, and the key knowledge will be repeatedly emphasized in the later classes.”	Well-organized course content and tasks	Students’ devotion and engagement	11	24
“This kind of assessment can enable us to engage in daily purposeful and directional study, instead of reciting and copying materials at the end of the term.” “Only by listening to the video carefully can we get a better score in the regular quizzes.”	Diversified course assessment methods			
“When discussing the case, you should think about the problem from an ethical perspective. This will deepen the impression of this aspect of knowledge.” “There were some cases in it, and after the knowledge was explained, I would repeat that case study.”	Case resources to guide memorization and reflection	Students’ in-class initiative	13	35
“Let’s say a case study, the teacher will guide us to think about it first, and then he will explain it.” “We have some case study classes, students are to make presentations, and then the rest asks questions, the teacher may help to answer from time to time. “	Student-centered approach to encourage reflection and interaction			
“It will be more helpful in the humanities piece. It embodies a kind of critical thinking.” “In the future, my approach to patients may be more comprehensive, and I will also consider the psychological and spiritual aspects of patients.”	Humanistic values in doctor-patient relationship	Students’ clinical decision-making capability	15	34
“After the class, I will be more integrated considering ethical and legal issues, while the way of thinking about the problem before was more flat.” “After finished studying, I would at least apply some principles to practice and would analyze what exactly should be done most appropriately from all aspects. “	Practical application-oriented teaching purpose			

## Results

### Theme 1: the influence of workload and assessment index on engagement

#### The arrangement of teaching content and memorizing and understanding key and crucial concepts

The main demand of blended teaching is to take the course syllabus as a whole and integrate pre-recorded videos and supporting online learning resources with in-person teaching organically and selectively [29]. Studies have demonstrated that the use of extracurricular (online) self-study to convey basic concepts and theorems, while focusing on difficult concepts in face-to-face teaching can deepen the level of learning and support internalization of knowledge [30]. This has also been the case in the present study. All 20 interviewees reported that the organization of the teaching content helped them learn purposefully and master the syllabus. Of these, 11 interviewees believed that the teacher could help students to understand, differentiate, and memorize the key and critical points more effectively by explaining the knowledge that they had encountered in prior self-study through case studies in the online class meetings.

*“It often starts with systematic theoretical viewpoints, and some specific knowledge points that still need to be supplemented by reading textbooks. The class meeting is mainly to help expand the perspective of thinking through deontology, consequentialism, basic ethical principles, etc., which are the backbone of the topic.”(Participant 3)*

*“The distinction of the key and difficult concepts, I think, is a little inadequate on video. The teacher talks more about those in class or mentions them or uses them in cases. I think they are probably what are called the key and crucial points.”(Participant 5)*

#### Diversified assessment strategies and comprehension ability

In blended teaching, it is necessary to deviate from the traditional assessment method used in Chinese education, which is solely based on the final mark, to address the teaching objectives and requirements. To comprehensively reflect upon the effectiveness of learning provided by online and offline classes, a combination of formative evaluation and summative evaluation should be adopted [31].

11 interviewees said that placing a higher weighting on their daily performance grade would act to circumvent the disadvantages of a purely exam-oriented assessment. While improving self-discipline through conducting daily learning activities, interactive modules can also substantially improve students’ comprehensive abilities.

*“This kind of assessment can help us to engage in daily purposeful and directional study, instead of reciting and copying materials at the end of the term. Moreover, writing a report can also test students’ ability to think critically, communicate and cooperate with each other.” (Participant 4)*

*“With marks given for daily activities, maybe students will be more active in preparatory and in-class performance, and the pressure on the final exam may not be so great.” (Participant 11)*

### Theme 2: the influence of resources and teaching on students’ initiative

#### Case resources to enhance thinking and learning

Adequate teaching resources are the basis of ensuring the smooth progress of blended teaching. The course included short videos, case analyses, test exercises, topic discussions, and other online content. According to the statistical analyses of the interview transcripts, the most highly rated resource was “case analysis”.

*“Cases are always more thought-provoking and leave a deeper impression, they give a more profound impression when thinking about the concepts.” (Participant 16)*

*“I feel that the cases are really novel and targeted. The dilemma of organ transplantation, for example, prompted profound reflection, as well as the trolley problem, which is helpful with learning to think critically.” (Participant 11)*

Such results may reflect that case studies and discussions are far more significant for medical ethics education than for other clinical subjects. As most medical disciplines fall within the natural sciences, teaching them is mostly driven by evidence-based concepts [32], where the evidence given leads to a particular conclusion or outcome. Medical ethics, by contrast, is the study of ethical issues in medicine, encompassing not only the rigorous concepts present in traditional medical disciplines but also the exploration of human and social relationships. Therefore, unlike traditional medicine, the answer to any medical ethics case is not straightforward or one-dimensional but requires a dialectical examination of the issue from multiple perspectives. Consequently, medical ethics education needs to give students opportunities to exchange and discuss ideas with each other to assimilate compound perspectives.

It is worth noting that case-study-based discussions typically benefit more from offline classroom instruction, where interactions between students and instructors are more natural through being face-to-face. Conversely, student discussion is more difficult to facilitate in an online meeting and the student experience is relatively weakened. However, where cases are presented using video and simulation technology, content may be more intuitive for students thanks to technological assistance. Therefore, during the COVID-19 pandemic when only online teaching was allowed, it was more important for teachers to consider how to maximize the advantages of electronic technology and choose cases appropriately. The availability of online teaching resources and the extent to which they are used will directly affect students’ enthusiasm for classroom participation and learning outcomes [33].

#### Student-centered classes encourage thinking and interaction

All in-class activities were designed to be student-centered, with the teacher only providing guidance. For example, the main purpose of the case study classes was to listen to students’ opinions, with the teacher contributing only to enrich the students’ understanding through eliciting responses and supplementing knowledge. All interviewees acknowledged that this interactive mode helped to stimulate their thought processes, which not only promotes in-depth communication between the teacher and students but also creates a consensus between viewpoints.

*“The class is student-centered. The teacher asks us to express our own point of view, which involves some common sense, not just the knowledge of ethics. He explores and expends around students’ knowledge.” (Participant 10)*

*“In online class meetings, the teacher lets us discuss the topic first, and then he summarizes and provides guidance.” ( Participant 11)*

In addition, students’ enthusiasm and interactions are also important indicators of the success of student-centered teaching methods. Student-student and student-teacher interactions reflect the appeal of a specific teaching method through behavioral and cognitive engagement [34].

*“In the live class, the teacher asks questions and encourages students to answer and to express their opinions. This is then done, and some students may refer back to this case to present another similar one, and then ask questions to the teacher, which is actually good.” (Participant 12)*

*“I am not the kind of person who is daring enough to speak in class, but I try to participate when I see other students doing so.” (Participant 15)*

### Theme 3: teaching orientation and clinical skills

#### Humanistic values ​​support the doctor-patient relationship

General Education is committed to cultivating the vision, complexity of thinking, independent personality, and critical awareness of students [35]. Medical ethics education is not only a general education but also cultivates skills in the professional humanities, such as the ability to care [36] and make ethical decisions [37]. When talking about whether the medical ethics course this semester has had an impact on their own values, the interviewees talked about learning and the impact on their future clinical practice. The impact on interviewees’ values ​​was mainly focused on paying more attention to the rights and interests of patients. The statistical analysis of interview transcripts showed that the frequency of words related to concerning patients/patients/doctor-patient, rights/interests, and communication occurred 25 times.

*“For example, now I will more carefully consider for the rights of the patient when it is necessary to expand the surgical zone in an operation.” (Participant 2)*

*“I will pay more attention to communication with patients in the future, I will notice that some of our actions may, while unintentional, cause the patients to become confused.” (Participant 13)*

#### Practical purpose orientation enhances clinical decision-making capability

Interviewees responded to questions such as “Through the study of the course this semester, do you think your application of knowledge and analytical capacities have been improved?”, and “Has this course been helpful for future clinical practice?”. Interviewees discussed their perspectives on matters such as the privacy of AIDS patients versus public health management, obtaining additional informed consent during an operation, and clinical ethical reviews.

*“I have been paying more attention to the privacy of AIDS patients, but now I may tend to think more in terms of public health, like the systematic management of AIDS patients using their real names. Medical institutions should also have records of these patients and pay attention to their movement.” (Participant 3)*

*“If there is any accident during an operation and it is not an urgent situation needing rectification, we should send a doctor from the operating room to communicate with the patients’ relatives, asking for their informed consent before deciding on how to continue the surgery. “ (Participant 6)*

*“I still worry about entering the clinical workforce, whether I can cope with the situation. But now I know that I can request an opinion from the hospital’s ethics committee immediately whenever I feel that I cannot handle the case by myself.” (Participant 5)*

As shown here, where there is a moral conflict, these students have demonstrated higher-order thinking skills such as those needed for criticism and reflection, and the analytical and comprehensive application of knowledge.

## Discussion

### Main findings

This exploration of the blended teaching model for medical ethics education was intended to make up for the deficiencies found in the previous practices of the SPOC and flipped classroom. In blended teaching, this study has demonstrated that the factors having the largest impact on the degree of course input and student engagement were the distribution of learning tasks and assessment models. Since blended teaching requires the integration of online pre-recorded content and in-person meetings, the key barriers to learning can be highlighted, students may become less bored with learning and may be less likely to forget what they have learned from self-study courses due to tedious course content or by topics being artificially split across sessions, and other barriers. Replacing summative assessment with formative assessment also helps promote daily self-directed learning and encourages active participation in classroom discussion.

This study also highlights the two most important factors affecting student enthusiasm for in-classroom learning; the availability of curriculum resources (especially case studies) and the teaching methods used. Using an Internet-based platform, we were able to maximize the use of various teaching resources inside and outside school and arouse students’ interest in learning by implementing a diversity of resources. Teachers’ repetition is no longer the main means of improving the retention of information, but the student’s interest in learning is stimulated and achieved through efficient knowledge transfer using case study analysis. In online class meetings, it is more helpful to stimulate students to express opinions with the teacher acting more as a guide rather than a leader. In the context of online courses including self-study, the main function of the teacher is also to provide immediate answers to students’ doubts, to train students to form correct ethical values and, to support their clinical abilities by improving their higher-order thinking skills.

### Limitations and directions for future research

Blended teaching is limited in its capacity to create opportunities for students to speculate and express opinions on ethical issues in terms of external objective conditions. At the subjective level, students’ enthusiasm and participation in the classroom are still closely related to their personalities and levels of experience. Although some students do not speak actively, they remain actively thinking. Students also learn by integrating the use of knowledge, analyzing case studies, and evaluating the views of other speakers. Due to the correlation between classroom participation and final course marks, 3 interviewees expressed their concerns about the bias of in-class interaction scores.

*“Some students may be more introverted and perform less well in class.” (Participant 11)*

*“I think many of the students who never spoke were actually listening to the lecture very carefully, but they were too shy to express themselves. This makes their scores differ greatly from those of their extroverted classmates, but in terms of their degree of learning engagement, I think they are similar in ability.” (Participant 16)*

As the subjects of this study were selected using a stratified sampling technique according to their final course scores, the sampling accuracy depended in part on the accuracy of the evaluation of student achievement using the online platform. Two interviewees reported that attention should be paid to the poor assessment results assigned in the online platform to activity such as meaningless comments in the discussion forum, and inaccurate supervision of video learning. These led to an inability to accurately differentiate student engagement with the online platform. Therefore, the supervision and management of the online platform must be improved with the addition of a personalized assessment facility, which can not only assist teachers from a technical point of view but also improve the accuracy of the assessment of teaching quality.

The limitations of this study highlight some interesting directions for future research. Although the definition of “learning effectiveness” has been kept as diverse as possible, interviewees’ dissatisfaction with the evaluation system is worth considering. As participant sampling was directly related to the measure of “learning effectiveness”, the reliability of this study is partially dependent on the accuracy of this metric. It is necessary to further develop and improve the diversity of assessment methods to adapt to different learning habits in the context of, and transition to, online learning, and to ensure that the true level of understanding can be more accurately reflected in students’ final results. Attendance, course engagement, participation, and test scores are all “objective” reflections of “learning effectiveness” based on teachers’ perspectives. However, rarely are implicit and imperceptible measures of effectiveness used, such as students’ sense of self-efficacy, improvements in learning motivation, and the expansion of knowledge in related fields. In higher education, self-report is often considered an effective indicator of students’ learning achievement [38]. Hence, questionnaire surveys and other tools to elicit students’ self-evaluation and feedback on learning can be introduced into future research. The credibility and accuracy of the data captured in studies such as this may be improved by assessing the variation between students’ subjective self-evaluation and objective final scores.

## Conclusion

Blended teaching methods implemented during the pandemic have been used as a compromise during periods when social distancing has been required. These methods have been shown to overcome limitations such as time constraints and room capacity limitations affecting the efficiency of teaching and learning. Using the example of the medical ethics course at Fujian Medical University, this study demonstrates the potential of a blended teaching format and provides an evaluation of the course by the students. This served not only to measure and address the shortcomings of previous online pre-recorded teaching and flipped classroom methods, but also to inform teachers how to approach planning course content, and organizing course activities and assessments, to accomplish the objectives of teaching.

## Supplementary Information

**Additional file 1.**

**Additional file 2.**

**Additional file 3.**

**Additional file 4.**

## Data Availability

All data generated or analysed during this study are included in this published article [and its supplementary information files].

## Abbreviations

FJMU: Fujian Medical University
MOOC: Massive Open Online Course
SPOC: Small Private Online Course
