# Supplementary material for: Blended teaching of medical ethics during COVID-19: practice and reflection
Source: BMC Med Educ. 2022 May 11;22:361. doi: 10.1186/s12909-022-03431-6 (PMC9094735; doi:10.1186/s12909-022-03431-6)
Supplement: Supplementary file 1 — Additional file 1. [file 12909_2022_3431_MOESM1_ESM.docx]

**Appendix**

**Question Set of the Semi-structured Interview**

1. The Medical Ethics teaching resources include digital resources in a variety of formats such as short videos of key concepts, case studies, test practice questions, and discussion topics. What do you think of these teaching resources? Can you give us an example?

2. The well-organized nature of the teaching content enables you to grasp the course syllabus as a whole, by integrating information from the online course lecture videos and supporting digital learning resources organically, and observing the key and difficult points. What do you think about the organization of the Medical Ethics teaching content in this round? Does it meet the above requirements for the organization of course content?

3. Do teachers pay sufficient attention to stimulating student participation during lectures? Do they involve students in explaining and commenting on cases as they go along? Does it contribute to student-teacher interactions? Is the teaching method student-centered? Can you illustrate this with examples from your own learning experiences?

4. After the lecture, do you notice any improvement in your competencies in skills such as critical and reflective thinking at the ethical level, the ability to synthesize and apply, the ability to analyze ethically, or other higher-order skills (please state)?

5. The course assessment method uses a combination of formative and summative assessments, do you think this method accurately reflects the ability of students? Is it fair and reasonable?

6. What do you think are the differences between the teaching you received in this course and traditional classroom teaching you experienced previously? What are the advantages and disadvantages?

7. Does the course achieve the fundamental objective of "building humanistic value"? Has it influenced your present and future development? Please expand on your answer by explaining your feelings.

8. Do you foresee any other issues with blended teaching using online resources? Can you suggest any improvements to the delivery of the course?
