# Supplementary material for: Blended teaching of medical ethics during COVID-19: practice and reflection
Source: BMC Med Educ. 2022 May 11;22:361. doi: 10.1186/s12909-022-03431-6 (PMC9094735; doi:10.1186/s12909-022-03431-6)
Supplement: Supplementary file 2 — Additional file 2. [file 12909_2022_3431_MOESM2_ESM.docx]

| **Fujian Medical University Course Teaching Schedule** | | | | | | | | | | | | | |
| --- | --- | --- | --- | --- | --- | --- | --- | --- | --- | --- | --- | --- | --- |
| Course: Medical Ethics | | | | | |  |  | |  | |  | |  |
| Class Hour Units：27 | | |  | | |  |  | | Total Enrolment：99 | | | |  |
| Class: Clinical Medicine Class 2018 | | | | | | | | | | | | | |
| Textbook: J.R. Liu and J.H. Yan, Medical Ethics. Wuhan：Huazhong University of Science and Technology Press, 2019. | | | | | | | | | | | | | |
| **Week** | **Date** | **Class** | | **Hours** | **Teaching Content** | | | **Teaching Space** | | **Teacher** | | **Notes** | |
| 1 | 2020-09-04 | 1-2 | | 2 | Ethics and medical ethics (face-to-face lecture, introduction to teaching requirements, case discussion; emphasis on analysis of the application, advantages and disadvantages of deontology and utility theory) | | | Online Meeting Class | | Chen Min | | The teaching design of the course is a mixture of online independent learning and online meeting class. Students learn basic theoretical knowledge through videos and other teaching materials on the online platform before each meeting class; in the meeting class, on the basis of online independent learning, teaching activities such as case analysis, group discussion and scenario simulation are carried out in combination with the teaching resources on the online platform to expand students' ability. After the class, the learning effect is tested through tests and assignments. | |
| 1 | 2020-09-04 | 3-3 | | 1 | Ethics and medical ethics (learn the basic theoretical knowledge, watch videos 1.1-1.4 and 2.1-2.4 of Medical Ethics on Chaoxingerya Platform; the important point is the understanding of basic views and basic theories of medical ethics) | | | Online Self-study | | Chen Min | |  |  |
| 2 | 2020-09-11 | 1-1 | | 1 | The normative system of medical ethics (learn the basic theoretical knowledge, watch videos 2.5-2.7 of Medical Ethics on Chaoxingerya Platform; the important points are the connotation of basic principles, norms and categories of medical ethics) | | | Online Self-study | | Chen Min | |  |  |
| 2 | 2020-09-11 | 2-3 | | 2 | The normative system of medical ethics (online course, ability development, case discussion; the crucial point is to be able to apply the basic principles and norms of medical ethics to guide and evaluate medical activities) | | | Online Meeting Class | | Chen Min | |  |  |
| 3 | 2020-09-18 | 1-1 | | 1 | Ethics of doctor-patient relationship (learn the basic theoretical knowledge, watch videos 3.1 to 3.4 of Medical Ethics on Chaoxingerya Platform) | | | Online Self-study | | Chen Min | |  |  |
| 3 | 2020-09-18 | 2-3 | | 2 | Ethics of the doctor-patient relationship (online course, ability development, case discussion; the crucial point is to analyze the rights and obligations of doctors and patients in the practice of diagnosis and treatment, and discuss causes of doctor-patient conflicts and possible adjustment methods) | | | Online Meeting Class | | Chen Min | |  |  |
| 4 | 2020-09-25 | 1-1 | | 1 | Ethics of clinical diagnosis and treatment (learn the basic theoretical knowledge, watch videos 4.1 to 4.7 of Medical Ethics on Chaoxingerya Platform) | | | Online Self-study | | Chen Min | |  |  |
| 4 | 2020-09-25 | 2-3 | | 2 | Ethics of clinical diagnosis and treatment (online course, ability development, case discussion; the crucial point is to guide students to analyze the ethical principles in clinical diagnosis and treatment in combination with the situation, and to understand the ethical requirements of diagnosis and treatment at different stages) | | | Online Meeting Class | | Chen Min | |  |  |
| 6 | 2020-10-09 | 1-1 | | 1 | Ethics of hospice care and death (learn the basic theoretical knowledge, watch videos 5.1 to 5.3 of Medical Ethics on Chaoxingerya Platform) | | | Online Self-study | | Chen Min | |  |  |
| 6 | 2020-10-09 | 2-3 | | 2 | Ethics of hospice care and death (online course, ability development, case discussion; the crucial point is to describe clinical situations to help students understand and master the application of ethics of hospice care and death) | | | Online Meeting Class | | Chen Min | |  |  |
| 7 | 2020-10-16 | 1-1 | | 1 | Public Health Ethics (learn the basic theoretical knowledge, watch videos 6.1-6.4of Medical Ethics on Chaoxingerya Platform; the important points are the theoretical foundations and ethical principles of public health ethics ) | | | Online Self-study | | Chen Min | |  |  |
| 7 | 2020-10-16 | 2-3 | | 2 | Public Health Ethics (online course, ability development, case discussion; the crucial point is to identify government actions involving the ethical application of public health, especially the handling of public health emergencies, through group discussions) | | | Online Meeting Class | | Chen Min | |  |  |
| 8 | 2020-10-23 | 1-1 | | 1 | Medical Research Ethics (learn the basic theoretical knowledge, watch videos 7.1-7.2 of Medical Ethics on Chaoxingerya Platform; the focus is on the requirements of medical research ethics and the connotation of ethical principles of biomedical research involving human beings) | | | Online Self-study | | Chen Min | |  |  |
| 8 | 2020-10-23 | 2-3 | | 2 | Medical Research Ethics (online course, ability development, case discussion; emphasis on setting clinical situations to help students understand and master the ethical requirements of research) | | | Online Meeting Class | | Chen Min | |  |  |
| 9 | 2020-10-30 | 1-2 | | 2 | Ethics of new medical technology research and application (learn the basic theoretical knowledge, watch videos 7.3-7.4 of Medical Ethics on Chaoxingerya Platform; the focus is on the requirements of medical research ethics and the connotation of ethical principles of biomedical research involving human beings) | | | Online Self-study | | Chen Min | |  |  |
| 9 | 2020-10-30 | 3-3 | | 1 | Ethics of new medical technology research and application (online course, ability development, case discussion; emphasis on setting clinical situations to help students understand and master the ethical requirements of organ transplantation and reproductive ethics) | | | Online Meeting Class | | Chen Min | |  |  |
| 10 | 2020-11-06 | 1-3 | | 3 | Medical Ethics Case Workshop (online course, ability development, case discussion; students work in small groups to present cases and discuss with the teacher and other students, with emphasis on a comprehensive examination of students' ability to apply knowledge, expression and collaboration） | | | Online Meeting Class | | Chen Min | |  |  |
