# Supplementary material for: Blended teaching of medical ethics during COVID-19: practice and reflection
Source: BMC Med Educ. 2022 May 11;22:361. doi: 10.1186/s12909-022-03431-6 (PMC9094735; doi:10.1186/s12909-022-03431-6)
Supplement: Supplementary file 3 — Additional file 3. [file 12909_2022_3431_MOESM3_ESM.pdf]

Question 1 regarding the teaching resources_word split.txt

Case/explanation/and/analysis/of/the teacher's/live class/There don't/seem to be/any/particularly/unique/resources/the online/courses/seem to/basically/include/these/modules.

Test questions/interspersed/throughout/the video/Students/will/be reminded/to stop/in the middle/stage/to help/them/focus attention on/the video/learning.

In fact/it feels like/the way/of/other/classes/It is/more/meaningful/that/the teacher/is discussing/some/key/and/difficult/problems/There are/also/case/discussion/classes/which are/quite/impressive.

The videos/of/the recorded/lessons/are/more/impressive/Because/there are/some cases/in it/after/explaining/the knowledge/I/will/study/that case/again/Then/the teacher/would/go over/it/again/during /the live/class.

I/think/it's good to/talk about/knowledge/in short video/class/because/it/get rid of/teachers/to talk about/rigid/knowledge/in class/and then/more/time/can/be left/for/case/analysis/which/will/make me feel/more impressed.

I/think/the live class/is/more profound/because/in fact/I think/many/teachers/will/find it/a little/troublesome/and so on/This/means that/we/can/ask/questions/directly/to/the teacher/during class/If there are/any/problems/it will be/more/convenient/to communicate with/each other/In addition/you can/ask/questions/in/the QQ group/and the teacher/answers/problems/in a timely manner.

I/feel that/the teacher's/discussion/in class/is/more impressive/In addition to/daily/videos/maybe/everyone/will participate/more/in the topic/discussion.

It should be that/there are/more/case/discussions/in/this course/compared with/other/short/videos/The videos of/other/courses/are/just/typing/things/from the textbook/on/powerpoint/and then/read/in front of/the powerpoint/in/the video/Then/the videos of/this/focus on/the knowledge/that is not/in/the lecture book/and more case/analysis/will/be conducted/Those previous/chapters/with role-playing/are more/focused on/narrating/cases/I/think/these/resource/on the network/has/a particularly/good/aspect/it will/separate/one/or several/chapters/to show/role-playing/and then/tell/the case/specifically/Because/there are/many/cases of/other/courses/either/only/talk for/a minute/or two/or/put/the course/case/in/the extra-curricular/materials/but/actually/we/are/generally/too lazy to/read/the extra-curricular/materials.

I prefer/case/discussions/During/the case/discussion/you/have to/think about/problems/from/the perspective of/ethics/then/and then/discuss/with/the group of/classmates/In this way/you will/deep en/the impression of/knowledge/in this area/Then/You/look at/the problem/from/this angle/and others/look at/it/from/another/angle/and/this problem/will/become/three-dimensional/So/I prefer/case /discussions/Although/it may/take more time/even/after discussing with/classmates/for an hour or two/there is no/result/For/the video/lessons/I feel/as if/I'm not/too impressed/Most of us/still/think that/the teacher's/live/class/combined with/some/cases/will be/more/intuitive /And then/it/will also/show/previous/role-playing/sitcoms/Although/I/know/how/they/want to/reflect/the contradiction /and then/how to/solve it/in fact /from/the beginning of/the case/I/can/probably/think of/the result /I felt that/it was/an established/routine/and/I felt that/I had not/get/anything/from/these/situations.

I/think/it's pretty good/I think/the most/impressive[profound/impression]/is/the melodrama/recorded /by/undergraduates/because/it intuitively/reflects/the scenes/like/medical-alarm/scene.

I/feel that/the cases/are/really/novel/and/targeted/The dilemma/case of/organ/transplantation/for example/led to/profound/reflection/as well as/the trolley/question/which/is helpful with/learning to /think/critically.

I/think/it's better/to combine/the cases/taught/by/the teacher/in the MOOC(Massive Open Online Courses)/with/the practice.
I/think/the video/is/very well done/The basic/knowledge points/are covered/and/the lectures/are/relatively/concise/If/I understand/all of these/thoroughly/there will be/no problems/in the general/exam/The/novel/aspect of/this class/is that/basically/every/class/is conducted/in the form of/case/discussion/The previous/classes/basically/taught/theoretical/knowledge/and/there is no/such form.
I/think/the materials of/Ethics/are completely/given to/us/and then/it is more/convenient for/us/to review/while/some/subjects/just/provide/powerpoint/or/some/knowledge points.
Because/many/courses/are/teaching/online/in this semester/Compared with/other/courses/Medical/Ethics/has/a different/aspect/from/others/in the learning /process/In fact/there are/many/things/that are not found/in textbooks/and there are/a lot of/expansion/I/think/this kind of/expansion/is necessary for/the ancillary/courses/If/you/just/read/books/it's/too/conceptual/and you /don't/get/as much/expansion of/knowledge/as online/teaching/What impressed/me/most/was that/when/I/saw/the online/video/I/saw/the case/that has been/co-produced by/students/and/teachers/in previous/years/and I/thought/it was/interesting.
I/saw/the online video/I/saw/the case/that/has/been co-produced by/students/and/teachers/in previous years/and/I/thought/it/was interesting/I/think/the difference from/other/courses/is that/the knowledge points/in the videos/are/quite/detailed/and the teacher/specializes in/analyzing/difficult/cases/in class/The content of/other/courses/is/more/repetitive/than that/of video/similar/content/I/think/Medical/Ethics/is quite/good/which is equivalent to/the teacher/lightening/some/burden on/us/and explaining/the difficult/points/What/impressed/me/most/was/the case/And then/In fact/I/have seen/some/cases/of previous/years/on/the platform of/the course/center/including/those/cases/that are/more/thoughtful/which/will have/a deep/influence/The knowledge points/that have to be/thought about/will be/more/impressive.
I/think/the teacher/speaks/clearly/in/the video/and/is/very/comfortable/to listen to/Then/there are/many/cases/in/each/class/We/are more/interested in/the case/which/makes/us/more/interested in/listening to/his/class.
What/impressed/me/very/deeply/was that/Ethics/could/have/a particularly/large number of/cases/and /these/cases/could/particularly/arouse/some/thinking /from themselves.
Some/online/video/in other/classes/are not/taken by /our own/teachers/and this/online/class/is taken by/our own/teachers/which/is more/targeted/Because/in terms of/exam/preparation/if/it is/the content/taught by/teachers /from/other/schools/you/won't be able to/determine/whether/it is/the key knowledge points/to be mastered/in /our/school/Moreover/our/teachers'/online/video/lectures/and/live/explanation/will be/more /continuous/The teachers/will be/more/clear about/what/they/have said/in/the video/lectures/and then/they/can/have/some/supplements/in/face-to-face/lectures.
It seems to/be similar to/the module/content/of other/subjects/The case/may/seem/a little/novel.

Question 1 regarding the teaching resources_word split_word-frequency (connotation). txt

Case/The case/cases 30
Teacher/the teacher/teachers/teachers'/the teachers' 19
Then/and then/moreover 15
Impression/impressed/impressive 11
Discuss/discussing/discussion/discussions 11
Course/the course/courses 13

Class/in class/taught 11
Knowledge points 8
Profound 7
Problem/problems 7
Ethics 5
Knowledge 5
Live 5
Study/learning 4
Analysis/analyzing 4
Explanation/explaining 4
Think about /reflect on/think/thinking/thoughtful/thought 4
Aspect 3
Novel 3
Perspective/angle 3
Role 3
Way 3
It seems to/it feels like/similar 3
Classmates 3
Expansion 3
Student 3
Playing/role-playing 3
Focus on/focus/focused on 3
Subjects 2
Understand 2
Teaching 2
Combine/combined 2
Interested in 2
The module/modules 2
Time 2
Convenient 2
Throughout/in the middle 2
Medical 2
Help/helpful 2
Extra-curricular 2
Textbook/textbooks 2
Compared with 2
Resource/resources 2
Intuitive/intuitively 2
Specializes/specifically 2
Repetitive/again 2
Chapters 2
Clear/clearly 2
School 2
Ask 2

Key 2
Scene 2
Targeted 1
Ancillary 1
Platform 1
On the network 1
Reflection 1
Continuous 1
Routine 1
Semester 1
Dilemma 1
Comfortable 1
Test 1
Previous years 1
Arouse 1
The general exam 1
Organ 1
Exam 1
Three-dimensional 1
Focused on 2
In terms of 1
Lightening 1
Too lazy to 1
Influence 1
Participate 1
Review 1
Stop 1
Theoretical 1
Detailed 1
Melodrama 1
Narrating 1
Transplantation 1
And so on 1
With each other 1
Established 1
Face-to-face 1
Practice 1
Burden 1
Probably 1
Each 1
Show 1
Led to 1
In a timely manner 1
Supplements 1

Actually 1

Center 1

Minute 1

Determine 1

Communicate 1

Thoughtful 1

Meaningful 1

Get 1

Hour 1

Group 1

Unique 1

Situations 1

Topic 1

Deepen 1

Concise 1

Put 1

MOOC(Massive Open Online Courses) 1

Rigid 1

Tell 1

Stage 1

Is equivalent to 1

Separate 1

Reminded 1

Daily 1

Trolley 1

Process 1

Co-produced 1

Interesting 1

Completely 1

The contradiction 1

Difficult points 1

Answers 1

Go over 1

Problem 1

Conceptual 1

Troublesome 1

Attention 1

Most of us 1

Be mastered 1

Question 2 regarding teaching organization and difficulties_word split.txt

It/was/fine/the course videos/are/rather/rushed/and/there/is/no way/to/fully/understand/the lessons/by myself/in/this/time/so/I/feel that/it/is better to/have/offline classes/where/the teacher/speaks/more/ intuitively/or/where/the class/is/a little/more/interactive/we/can/distinguish/between/important/and/

difficult/points.
It/was/fine/the online course/lecture/videos/have/too/little/knowledge/compared to/the book/Feels like/the course/focuses on/doctor-patient/communication/as/it/is more/clinical/the difficulty/lies/in the dilemma cases/for which/no clear answers/can/be given/for example/decisions/on/doctors'/ behaviour/that/are/not/regulated/by law/for the time being.
I/feel that/there is/still/something/there/because/from/the beginning/it/feels/more/systematic to/talk/ about/some/ideas/and/theories/and then/specific/problems/and/analysis/and/overall/it/can/still/solve/ the problems/that/may be/encountered/in real life/However/it/may/be necessary to/combine with/ the textbook/and/some/specific/knowledge points/still/need to/be added/through/the textbook/the teacher's/lessons/are/more/helpful/in/broadening/the perspective of/thinking/the point/is/that /deontology/consequentialism/fundamental/principles/Because/these/are/the backbone/the clues/that/ lead us to/the case/studies/But/it/is possible that/what/you/think/is/the focus/now/will/be understood/differently/later/when/you/study/it/in depth.
I/thought/it/was/quite/good/and/felt that/all aspects/topical/social/issues/and/medical/aspects/were more/comprehensive/the teacher/starts/the class/b/explaining/what/needs/to be mastered/and/what/ just/needs/to be/understood.
It/should be/said that/it/is/very/possible/In fact/the distinction of/the key/and/difficult concepts/I/ think/seems to be/a little/inadequate/on/video/the teacher/talks/more/about/those/in class/or/ mentions/them/or/uses/them/in/cases/I/think/they/are/probably/what/are/called/the key/and/difficult/ points.
I/think/I/will/have/a/better/concept/of/medical/ethics/as a whole/and/every/lesson/is/relatively/clear/I/ think/so/because/our/courses/are/a complete set/but/because/it/may not/be necessary to/learn/all/the courses/in/the syllabus of/our/major/then/the teacher/said/which/courses/are required/and/then/the re st of/the courses/are/expand/and/the teachers/who/focus on/mastering/them/have/clear/instructions/If /there is/something difficult/when/the teacher/is/in class/he/will/explain more/and then/the important points/will/be/repeatedly/emphasized/in/later/classes/I/think/I/can/feel/it/by myself/I/think/ it/is/mainly/reflected/in/the live class/because/some/knowledge points/will/be/mentioned/constantly/ by teachers/and then/I/will/feel that/this/is/very/important/for example/the contradiction/between/ the patient's/autonomy/and/the doctor's/right/to/special/intervention/the teacher/said/a lot of/cases/It/ is particularly/impressive.
I/think/there is/basically/Teachers'/lectures/are/usually/the key points/the emphasis/should be/on/the /theoretical part/of/the previous chapter 2/because/it/is/more/suitable/for the exam/But/I/don't/have/ my/booksath/and/now/and/I/feel like/I've/forgotten/all/about/them/But/the teacher/is not/testing/our/ ability/to master/knowledge/but/giving/us/an/insight/into/what/you/want/to do/clinically/and then/ why/you/do/it/which/makes me/very/impressed.
I/think so/but/the after-school exercises/in/small/chapters/are/repeated/with/some/after-school/ exercises/in/big/chapters/and/there/are/quite/a lot of/repetitions/I/didn't/feel/it/reflected/the important/ and/difficult/points/the emphasis/should be/on/the previous/principles/Because/they/run/through/the later/doctor-patient/management/In fact/these/principles/are/reflected/in/the later chapters/so/I/think/ it/should be/the key/point/and/every time/a/case/is/analyzed/the teacher/will/start/from/these/parts.
The order of/our/classes/at that time/generally/taught/some/basic/theoretical/knowledge/first/and then/the later theories/were divided/into/different/sections/such as/public/health/and/reproductive/ ethics/this/is/a/fairly/common/learning/system/the principles/to be followed/mentioned/in/the following sections/are/actually/some/what/repetitive/with/those/mentioned/in/the previous/general/

introduction/Although/there/are/repetitions/It/is not/exactly/the/same/and/each/chapter/has/these/ principles/so/it/is/a bit/easy/to/mix up/and/this/is not/the test site/When/you/take/the exam/you/also /know that/the hard points/are/a bunch of/principles/mentioned/in/the book/Most of/the questions/ in/the exam/are/asked/you/to/think/ethically/which/is/also/about/the application/of/those/principles/ then/I/think/the difficulty/may be/that/some/principles/are/to o/similar to/distinguish/them/After all/ when it comes to/dealing with/exam/questions/there/are/still/mechanical/memories/as well as/pure /thinking/for example/the public/health/chapter/seems to/be/of/little/importance/and/there/is/no/PPT/ this/part/is/occasionally/tested/once/or/twice/In addition/the teacher's/PPT/overlaps/with/the/uploaded /video/which/I/think/should be/the focus.

Yes/because/I/think/the teacher's/speech/is/quite/prominent/for example/the second/chapter/of/ deontology/is/obviously/the key point.

Teaching/is/definitely/organised/It/is/gradually/progressive/according to/the difficulty/of/each/chapter /and/fully/explained/there/are/also/some/sitcoms/in/the teaching video/which/is/a/good/way/the teacher's/PPT/will/mark/the important/and/difficult tips/In addition/we/have/a/syllabus/for/the content/that/the teacher/focuses on/in/the video.

Yes/because/it/has/many/modules/Including/doctor-patient/relationship/ethics/medical/frontier/ethics/ On/the Chaoxingerya/It/is/also/taught/in/modules/each/part/starts/with/the general theory/then/talks/ about/several/basic/principles/and then/divides/it/into/some/sub-modules/I/think/it/should be/quite/ beneficial/Regarding/the difficulties/of/teaching content/what/the teacher/mentioned/in/the live class /may be/more/required/to be/mastered/what/we/learn/on/the learning platform/should be/most of/ the conventional/knowledge points/but/I/think that/if/there/are/important/and/difficult points/It/ should be/reflected/through cases/or/what/the teacher/has/said/Generally/which/module/is/discussed/ in/the case/which/module/is/more/important.

I/think/there is/the whole course/starts/from/the first ethical principles/and then/is/precise/to/all/ aspects of/ethics/In/this/way/I/think/the whole framework/should be able to be/grasped/Regarding/ the difficult/points/of/knowledge/I/don't/seem to/feel/much/But/for/the key points/I/think/the basic/ principles/and/basic/view points/of/ethics/taught/in/the first/few/lessons/are/the/most/important/and/ the rest/of/the content/actually/revolves/around/these.

I/think/it/is/well/organized/First of all/the teacher/has/developed/the syllabus/according to/which/we /can/study/there/quired/knowledge points/Secondly/the teacher's/teaching/video/has been/posted/on/ the platform/and/we/can/watch/it/the last/and/most/important/point/is that/the teacher/interacts/with/ the students/during/the class/Including/answering/the questions/and/talking/to students/I/think/this/ way/contributes to/the absorption/and/application/of/knowledge.

Yes/I/think/it's/all/very/organized/I/seem to/remember that/the content of/the first/and/second chapters/is/partially/theoretical/and/each of/the following ones/is similar to/the specialty/and/small/ topics/After/learning/and/comprehending/the content of/the first two chapters/and then/applying/it/ to/each of/the subsequent/sub-disciplines/I/feel that/it/is/very/organized/In fact/I/don't/think/I/have/a/ very/good/understanding/of/the difficult/and/important point/in class/but/the teacher/has mentioned/ some/key points/in class/and/I/have/seen/it/when/I/take/notes/but/for/my own/later/review/I/still/ follow/my own/feelings/because/I/think/when/I/learning/these/It/must/have/been/for/future/clinical/ use/therefore/I/will/focus on/what/kind of/problems/I/will/encounter/in/clinical/practice/in/the future/ or/what/aspect/I/am/interested/in/and/experience/them.

The short video/of/the knowledge point/for/the statement/of/the case/coupled with/an/in-depth analysis/of/the case/by the teacher's/live class/complements/each other/I/think/the focus/should be/

on/the basic/principles/and/categories/of/ethics/in/the/second/chapter/which/is equivalent to/the entire/framework/of/ethics/and then/in/the specific/explanations/later/each of/them/is based on/the previous theory/As for/the difficult part/I/think/chapter/8/new/technology/research/is/difficult/ Because/I/think/ethics/is/more/inclined to/logical/analysis/such as/interpersonal/relationship/ethics/ and soon/I/can/straighten out/the logic/and/think/how/this/matter/should be/handled/but/the new technology/I/am more unclear/I/feel that/what/ever/you/do/It/seems to/be not/very/good/Because/it/ is not/like/other/cases/such as/interpersonal relationships/which/you/just/think/about/the relationship /between/A/and/B/It/needs to/think/about/the impact/on/the whole human/society/and/the impact/on /the future/and/it/'s/very/broad.

If/you/watch/the video/alone/you/still/don't/feel like/you/have/a/holistic/feeling/then/after/listening to /the teacher's/series of/lectures/there is/more/overall/understanding/I/don't/think there/are/enough/ test/questions/I/usually/want to/find/some/questions/to answer/to test/the effect/after/learning/but/it/ is/more difficult/to/find/I/usually/look at/those/existing/there/are/some/problems/that/teachers/need to /discuss/in class/which/can/be difficult/for example/should/we/consider/social/or/medical/factors/for/ organ/donation?

I/think/it/can/still/the key/and/difficult point/because/I/remember/a teacher/said/textbooks/in/the second chapter/is/the key/video/is/more/I/remember/the chapter/looks difficult/also/will/let/me/spend /more/time/to/think/behind/such as/artificial/in/semination/clinical/medical/technology/of/public/ health/in/these/cases/I/think/is not/the key/and/is/relatively/easy/to understand/So/it/feels like/the point/is/still/quite/prominent.

I/think/it's/fine/In fact/I/think/the time/is/relatively/short/there/must be/some/places/to/focus on/I /think/it/'s/quite/clear/that/the first ethical principles/should be/the focus/and then/things/like/patient /voluntary/should be/the difficult/ones/because/it/can be/combined/with/a lot of/clinical/examples

I/think so/In/the PPT/given/by/the teacher/there/will/be/a/detailed/mark/out/which/are/the key/points /to/master/He/would/also/say/before/the class/what/to know/what/to be/familiar/with/what/to know /these/are/already/distinguished/It/is/the public/health/capacity/that/is/more/difficult/to make/decision s/because/it/is/more/theoretical/and/targeted/at/the macro level/especially/considering/the interests of /society/the emphasis/is/on/the relationship/between/clinical/treatment/and/the patient.

Question 2 regarding teaching organization and difficulties_word split_word-frequency (connotation). txt

Teacher 28
Then 22
Important points/tips 21
Difficult points/tips 16
Chapter 16
Ethics 13
Principles 12
Cases 11
Study 10
Clinical 7
Knowledge points 7
Mastery 7
Courses 6

Teaching	6
Question	6
Front	6
Attend/in class	6
Medicine	5
Said	5
Analysis	5
For example	5
Examinations	5
Learning	5
Start	5
Whole	5
Reflections	4
What	4
Above	4
Theory	4
Outline	4
Module	4
Public	4
Distinction	4
Repeat	4
Overall	4
Health	4
Second	4
Mentioned	3
Emphasis	3
Comprehension	3
Relationships	3
Expand	3
Social	3
Outstanding	3
Applications	3
Foundation	3
Time	3
Technology	3
Local	3
Emphasis	3
Live class	3
Textbooks	3
Doctor and patient	3
Capabilities	3
Remember	3
Patients	3
Clarity	3

Mutual 2
Principle 2
Knowledge 2
Perspectives 2
Framework 2
General remarks 2
Description 2
Discussion 2
Sitcom 2
Impressions 2
Clear 2
Angle 2
Morality 2
Approximate 2
System 2
Comprehension 2
Title 2
Each 2
Each time 2
Familiarity 2
Processing 2
In depth 2
Supplementary 2
Decision making 2
Logic 2
Exercises 2
After Class 2
Completely 2
Books 2
With points 2
It says 2
Doctor 2
Combined 2
According to 2
Factors 2
Interpersonal relations 2
Answer 2
Impact 2
On the one hand 2
General 1
Around 1
Macro 1
Awareness 1
Deficiencies 1

Thinking 1
Repeatedly 1
Encounter 1
Most 1
Memories 1
By me 1
Progression 1
Consequences 1
Rules and Regulations 1
Tell 1
Reality 1
Weekdays 1
Platform 1
Follow 1
Frontier 1
Available 1
Organisation 1
Testing 1
Tendencies 1
Things 1
Category 1
Interests 1
Beneficial to 1
Organs 1
Exam Points 1
Examine 1
Examinations 1
Concepts 1
The Future 1
Subjects 1
Details 1
Hold 1
Interests 1
Adequate 1
Encounter 1
Classes 1
Comprehensive 1
Testing 1
Suitable for 1
Voluntary 1
Application 1
Examples 1
Management 1
The vast majority 1

Review	1
A complete set	1
Rationalisation	1
Rational	1
Subsections	1
Communication	1
Divided into	1
Down	1
Backbone	1
Slightly	1
Dismissal from class	1
Take your time	1
Hotspots	1
Special	1
Obviously	1
Experience	1
Humanity	1
Departure	1
Interaction	1
Common	1
Two Difficulties	1
Machinery	1
Large mould	1
The Law	1
Behaviour	1
Temporary	1
Slightly	1
Forget	1
Theorising	1
What is called	1
Time will	1
Classmates	1
Deep	1
Specialties	1
Theory Department	1
Notes	1
So	1
Interventions	1
Method	1
Artificial insemination	1
Donation	1
Difficult to find	1
Subdivision	1
Throughout	1

Research	1
Precise	1
Specification	1
Provisions	1
Conversation	1
Help	1
What to see	1
Reproduction	1
After all	1
Explanation	1
Social	1
Frequently	1
How	1
Equivalent to	1
Perception	1
Compared to	1
Close to	1
Absorption	1
Difficulty	1
Direct view	1
Plus	1
Effects	1
Autonomy	1
What kind of	1
Treatment	1
Clues	1
There are articles	1
First	1
Difficult	1
Once or twice	1
Benefits	1
Overlap	1
Similar	1
Order	1
Contradictions	1
Remaining	1
Able to	1
Unlike	1
Presentation	1
Not in	1

Question 3 regarding student-centred_word split.txt

Yes/there/is/When/explaining/the cases/the teacher/will/say/that/if/you/have questions/you/can/

always/ask/them/and/discuss with them/and/then/the teacher/usually/will/show you the answers/ which/will/help/us/more than/asking the teacher/after class/The teacher/is/more inclined to/let/the students/think about/it.
Yes/there/is/The teacher/asks/the students/if/they have/any comments/during the live classes/ and/in the final/case discussion course/we/are/basically/discussing/all the time.
In fact/I/am/just/speaking personally/I/basically/didn't/answer the questions/usually/the main thing/is/to listen/But/we/all/think/and/maybe/for those students/who/are/more active in speaking/they/may/be able to/express themselves/The effect/of the interaction/felt/similar to/ offline/If/we/look at it/in terms of time/basically/the teacher/is/still/the centre/Because/the course/may/be/tight for time/and/may not/be/so in-depth/when/the teacher/is/explaining/he/may not /be/so in-depth/to/dig out/the students' ideas/and/summarize them/with/a little bit/But/it/is/also/ inspiring/If/I/had/more time/or/more opportunities/I/would/have been/involved in/the discussion.
Yes/We/would/be asked to/analyse/the case/ourselves/too/and/then/make a PowerPoint presentation /to/explain it/After/listening to/our students' presentations/the teacher/will/have a commentary/at the end/When/The teacher/is/explaining/the case/there/is/an/opportunity/for/the students/to/ participate in/it/and/comment on it/while explaining/There/is/facilitated/interaction/Teaching resources/are/given to/us/The/PPT/also/has/a clear focus/and/we/are given/guidance on/how to analyse/the case/and/then/we/are allowed to/discuss it/on our own/and/we/can/express/any different opinions.
Actually/there/is/In the case of/a case/The teacher/will/guide/us/to think first/and/then/he/will/ explain/For example/what/ethical principles/are involved in/this/case/or/if/you/are/a doctor/what/ should/you do/to/meet up with/the ethical requirements/the most/and/then/ask/the students/to answer/Because/now/during the epidemic/I feel/that/there seems to be/less communication/ between/my classmates/The/interaction/between/students/and/teachers/supposed to/be/good/but/after all/the duration of the course/are/limited/Maybe/only/some students/can/communicate with/ teachers/In terms of/student-centered/I/think/it/would/be/much better than/other courses/and/much better than/other courses/that/focus on/knowledge.
Yes/during the live class/The teacher/would/stop/and/ask/us/what/problems/we have/When/ everyone/has finished/asking questions/and/he/has finished/answering them/he/will/continue to/ move on/I/think/in this way/the learning memory/will/be/deeper/Because/some students/will/help/ you/ask/the questions/you/want to/ask/In this way/although/you/don't ask/you/will/remember/more deeply/in the process of/listening/In terms of/student centered/I/think/The teacher/still/attaches great importance to/what/problems/we/will/encounter/in clinical practice/in the future/He/will/often /give some clinical examples/and/then/pay great attention to/whether/we/keep up/or/do not/ understand/This/should/be called/student-centered/In addition/we/have/two/case analysis classes/all of students/will/display cases/by ppt/then/other students/ask questions/and/then/the teacher/answers /them.
Yes/there/is/But/I/think/the atmosphere/would/be more intense/at school/I/think/the teachers/have/ brought them up/all/Generally/The teacher/will/lecture/some real information/first/and/then/we/will/ discuss/the case/Everyone/will/express/their opinions/and/then/The teacher/will/come back/and/sum marize.
Yes/Basically/in every class/whenever/there/is/a case analysis/the teacher/always/asks us/to express our opinions/and/will/put forward/some opinions/and/suggestions/of his own/ according to/our opinions/It/promotes/(student-teacher)interaction/In fact/sometimes/when/I/hear

about/that/case/I/explode not without reason/or/there are/some questions/at the moment/If/we/are/ asked/to give our opinions/after/a class/is finished/it/is/possible/that/we/have forgotten about/it/or/ just/remembered/a little/question/There/is/student-centered/he/will/emphasize/that/if/we/don't understand/we/can/ask/them/immediately/and/The teacher/will/give us/a serious explanation/of our questions.

I/think/the teacher/is/working hard/to do these things/but/everyone's participation/may not be/very high/For example/he gave a case/and/then/he/would/ask about/our thoughts/After/one or two classmates/had answered/he/would/also/ask/if anyone else/had a different point of view/but/not many classmates/participated/Because/at home/classmates/will/be/more relaxed/than/in class/and/ they/may/be doing/other things/while/in class/such as/playing games/In class/I/think/more/people/ will/listen carefully/and/then/more/people/will/participate in/this way/Also/when/there/is/a big discussion/in class/everyone/talks to/each other/In this situation/students/will not/have/too much/ pressure/to/participate in/the discussion/However/the speeches/in the online class/were/one by one /The shy students/may/be afraid/that/their statements/will be refuted/by/others/If/they/have been discussing/all the time/it/is/basically/not clear/who/said/it/Basically/during the discussion/the teacher/will/ask/us/what/we think/and/if/there/is/a different method/we/will/put it up/again/but/there seems to be/little communication/between/the classmates/The teacher/will/always/listen to/our opinions/firstly/Generally speaking/we/will/mention/the few points/that/The teacher/wants to say/ and/then/The teacher/will/confirm/what/we/have mentioned/firstly/and/then/add/something/that/we/ have not mentioned/or/provide/another way/of thinking/I/think/this way/is/good/In fact/students/ also/want to/know/some things/they/didn't expect/or/didn't know/in class.

Yes/I/think/there/is/I/feel/that/some students/are/still/embarrassed/to speak/Maybe/the surrounding environment/is/noisy/I/think/the discussion/in the meeting class/may/be/better/Even so/the teacher/ has/always/encouraged/the students/to actively speak/in class/I/find/it/useful/Communication/can/ lead to/a collision of ideas/and/better/ideas/As/it/is discussed/online/this effect/may/not be/so good /The class/is/student-centered/Just like/at the beginning of/the course/there/was/a case of/two children/arguing about/the sun/and/then/The teacher/asked/us/to express/our own/point of view/ which/involves/some common sense/from/daily life/not/just/the knowledge/of ethics/He/explores/ and/expends/around/students' knowledge.

In the live class/the teacher/asks/some questions/and/encourages/students/to answer/and/to express /their opinions/We/all/have done/it/and/some students/may/rely on/this case/to present/another/ similar one/and/then/ask questions/to/the teacher/which/is/actually/good/Of course/this form/can/ promote/interaction/but/after all/this/form of/class/is/relatively special/and/the effect/is/ definitely/not/as lively as/the meeting class/discussion/In/live class/meetings/the teacher/lets/us/ discuss/the topic/first/and/then/he/summarizes/and/provides/guidance/Maybe/I/am/still/less/ involved/because/I/am/a bit/shy/so/I/speak/less/online/I/may/be/more/involved/in/large-scale discussions/but/when/the teacher/asks/a few/students/to/speak alone/I/am/less/involved.

Yes/The teacher/will/interact with/us/during the live class/and/ask/us/to talk about/our views/on the case/I/think/there/is/still/a way/for/students/to participate in/commenting on/the case/ Because/the teacher/mainly/gave/us/a general direction/and/then/let/us/take/the initiative/to/ answer questions/about/knowledge points/I/think/this/method/will/definitely/promote/interaction/but/ the premise/is/that/you/must/be able to/consciously/complete/some/teaching videos/of/the learning platform/and/have/a little/impression/of/the knowledge points/before/you/can/apply them to/these/ specific cases/Therefore/the ability/of/self-learning/is/more important/before/this/otherwise/you/will/

not/know/what/The teacher/is/talking about/I/think/this method/must/be/student-centered/because/ teachers/will/inspire us to/think about/problems/be/more empathetic/and/be able to/think/in an empathy/He/will/look at/what/problems/may/be easier/to understand/from/the perspective/of student s/and/then/talk about/the problem/intuitively/I/think/these/forms/make/it/easier/for/us/to understand.
Questions/are/often/asked/during/class/Usually/students/are/not involved in/the case explanation/ during/the meeting class/but/our last class/was/a group case discussion/with/everyone/participating /The teacher/will/interact with/the students/in class/ask/some questions/and/then/continue/to explain/This method/will/make/students/learn/faster/and/it/will/definitely/be better/than/simply/ listening to/the teacher/During/the class/the teacher/often/said/that/anything/you/don't/understand/ you/can/ask/again/or/you/can/continue to/discuss/after/class/When/we/answer/questions/students/ always/express/their/own views/and/then/teachers/also/briefly/comment on/the shortcomings/or/ limitations/of/their views/because/ethics/does not/have/a/fixed/answer/I/think/everyone/can/express/ their/own/opinions.
Yes/there's/a lot of/discussion/because/everybody/wants to/speak up/The/interaction/between/ teachers/and/students/does/exist/because/we/keep/discussing/all the time/We/would/discuss/some ethical issues/in/our/online study group/with/each other/We/discuss/anonymously/in the group/so/ we/often/ask/discuss/consult/The teacher/and/so on/I/think/the teacher/has/a/good/habit/that/he/ would/allow/students/to interrupt/at any time/after/finishing/the illustration/of/a case/and/then/he/ would/discuss with/us/You/can/ask/during/the class/or/after/the class/if/you/don't/understand/the points/I/think/it/is.student centered.
Yes/the teacher/would/ask/some questions/in class/and/I/am/not/the kind of/person/who/dares to/ speak/in class/but/I/look at/the participation/of/other students/and/then/I/follow/the teacher's thinking/and/I/think/the degree/of/participation/is/quite high/Teachers/usually/show/us/cases/first/and/ then/let/students/speak freely/In fact/we/can/also/see/the viewpoints/of/different students/ their emphasis/is not/the same/If/you/put together/so many views/you/can/actually/sort out/an answer/that/is/close to/the teacher's/As for/student-centered/online courses/are/difficult/because/its/ can't/interact with/teachers/face to face/or/have/a long time/to/ask questions/after/class/like/we/do/ in school/Therefore/if/they/are/student-centered/even/the meeting class/are/quite/difficult.
Yes/Because/The teacher/talks about/the key points/through/cases/it/is/necessary/for/everyone/ to participate in/the discussion/and/express/their views/Yes/When/The teacher/is/speaking/if/the students/have/problems/or/ideas/they/can/speak directly/or/type/so/they/can/discuss/in the same time /Of course/there/is/and/it/will/obviously/be/much better than/other classes/because/the hardest part/of/online teaching/is/to be interactive/Most of/the other courses/are/lectured/by teachers/but/different/students/in this class/will/have/different/views/and/students/and/teachers/ must/communicate/and/then/they/also/have/different/views/between/students/which/is/actually/a collision of/views/Because/after/coming up with/a case/the teacher/does not/explain/first/and/ guide/the students/on/how/to do/it/Instead/it/is/to/come up with/a case/and/then/the teacher/ directly/lets/us/analyze/it/ourselves/and/then/explain/it/on the basis of/our analysis/I/think/one of/ the things/that/the teacher/is/particularly/good at/is/that/in general/he/will/affirm/our/point of view/ first/and/then/he/will/add to it/in/more/respects.
The teacher/often/let/us/open/the wheat/by ourselves/and/any questions/can/be raised/to discuss/and/The teacher/will/also/give/us/answers/in time/It/helps/If/it/is/a/traditional/class/ students/may/not/answer/and/ask questions/so/enthusiastically/If/you're/online/maybe/people/are more willing to/ask questions/I/think/our/group paper discussion/at the end/was/quite/facilitated/

Before/we/thought about/it/ourselves/the group/also/discussed/a lot of/directions/and/then/during/the period/we/will/take the initiative to/ask/the teacher/whether/this case/is/ok/or/not/After/getting/ the teacher's affirmation/we/firmly/do/it.
Yes/Because/most of/the students/may/slack off/during/the/live class/and/then/these students/ who/have listened/carefully/may/feel/bored/without/participating/and/they/will/hang over/there/ later/and/may/not/listen/But/asking questions/in/this/kind of/discussion/that/we/can/all/participate in/ will/allow/students/to/go deeper into/the/course/and/have/a/deeper/understanding/of/the case/Student -centered/in fact/there/are/many/aspects/now/on the one h/and/the teacher/also/considers/for/our students/for example/we/finally/had/a/case sharing/session/but/because of/limited/time/it/is/ impossible/for/every/group/to/participate/and/The teacher/said/that/whether/he/participated in/the sharing/or/not/he/would/look at/the content/of/the paper/and/everyone's score/was/not/so bad/For e xample/the teacher/often/throws/the case/to/us/open-ended questions/let/us/answer/first/and/ then/he/will/summarize/our views/and/the/words/of/the students/next to us/may/continue/to/add/and/ then/The teacher/will/continue/to summarize/and/including/the good/and/bad/points/we/talk about/ will/be summarized.
I/think/it/is/more/obvious/in/the/live class/analyzing/some cases/with/us/which/will/lead/us/to/think about/such/a/process/In fact/The teacher/encouraged/us/but/there/were/not/many/people/spoke/ actually/but/even though/we/did/not/speak/we/would/think/in/our/own mind/first/and/finally/ compared to/the/opinions/put forward/by/the teacher/Interactive/is/helpful/Because/the/video class/ may/not/be/so/targeted/because/it/is/recorded/after all/we/also/need/to/make a list of/rules based on/ the teacher/we/figured it out/and/then/when/on/the live class/that/is/student-centered/generally speaking/these/cases/are/very/interactive/In the beginning/The teacher/will/introduce/knowledge points/intersperse/cases/and/then/think about/how/to/deal with/them/Finally/there/will/be/a summary/ Although/teachers/have prepared/lessons/in advance/in class/they/can/make/targeted adjustments/acc ording to/some feedbacks/and/answers/in class.
Yes/Communication/between/students/and/teachers/should/be facilitated/but/only/by/a few students/who /speak up/We/used to/do/group case studies/and/we/would/build/our/own/group discussions/I/think/ The teacher/generally/provides/us/with/a/way/of/thinking/in class/and/then/asks/us/more/about/our think ing/about/those questions/because/he/often/asks/us/what/do/we/think/so/I/think/if/we/listen to/it/carefully /we/will/think about/these/questions.

Question 3 regarding student-centred_word split_word-frequency (connotation) .txt

Teachher 71
Students 64
Then 48
Question 33
Case31
Discussion 21
Having class 16
Answer 16
Class 16
Speaking15
Perspectives15
Interactive 13

Center 12
Lecture 12
Participate in 11
Thinking 10
Put forward 9
Analysis 8
Opinion 8
Often 8
Promote 7
Time 7
Communication 6
Conclusion 6
Deep 6
Place 5
Like 5
Live 5
Understand 5
Ethical 5
Focus on 5
Group 4
Process 4
Carefully4
Can 4
Idea 4
After class 4
For example 4
Effect 3
Teaching 3
Study 3
Add 3
Express 3
Knowledge point 3
At any time3
View 3
After all 3
Discuss 3
Encourage 3
Over 3
Collision 3
Published 3
Paper 3
With 3
Clear 3
Intense 2

Present 2
Do 2
Shy 2
How about 2
Involved in 2
Warm 2
Limitations 2
Meet 2
Inspired 2
Initiative 2
Most 2
Clear 2
Guide 2
Mentioned 2
Targeted 2
Form 2
Participation 2
At ordinary times 2
Previous 2
Help 2
School 2
Don't understand 2
Things2
Report 2
Positive 2
Clinical 2
Almost 2
Knowledge 2
Practice 2
Point 2
Part 2
Remember 1
Same 1
Memory 1
Information1
Big 1
High 1
Etc/1,
Sometimes 1
Free 1
Self-study 1
Consciously 1
Independent 1
As 1

Rely on 1
Around 1
Entity 1
Suggestion1
Character 1
Real time 1
Any 1
Adjust 1
Score 1
Thought 1
During the period of 1
Side 1
Close to 1
Hear 1
Run into 1
Discover 1
Complete 1
What kind of 1
Summary 1
Talk about 1
Ask 1
Boring 1
Premise 1
Type 1
Interrupt 1
Anonymous 1
Face to face1
Less than 1
Allow 1
Environment 1
Comment on 1
Scope 1
Otherwise1
Direction 1
Doctor 1
Feedback 1
Counter 1
Method 1
Empathy 1
Example 1
Followed 1
Guide 1
Part 1
Principle 1

All right/1

Application 1

Tend 1

Home 1

Stop 1

Sort out 1

It is better to 1

Should 1

Down 1

Priority 1

Than 1

Insights 1

Common sense 1

Deal with 1

According to 1

What 1

Opportunity 1

Prepare 1

About 1

Based on 1

Each 1

Outbreak 1

Open 1

In time1

Emphasis 1

Explanation 1

Internet 1

Above 1

Guide 1

Said 1

Arrange 1

Make 1

Image 1

Special 1

Firm 1

Efforts 1

Exploratory 1

Children 1

More than 1

It is better to 1

In heart1

Compared to 1

On the one hand, 1

Rules 1

Each hold 1
Get 1
Value 1
Simple 1
Pressure 1
Relax 1
Expand 1
Produce 1
In advance1
Just 1
Alone 1
Resources 1
Intuitive 1
Farewell 1
Atmosphere1
Ability 1
Game 1
Homecoming 1
Return 1
Splack off 1
Excuse me 1
All 1
Come 1
Fixed 1

Question 4 regarding ability Improvement_word split.txt

There/must/be/some/but/not/too much/depth/The view/of/the case/will/be/a/combination/of/ethical/and/legal issues/whereas/previously/the/way/of/thinking about/the issue/was/flatter/An/example/is/the issue/of/organ transplantation/at the time of/the group case/report/An/18-year-old/boy/with/an/intellectual/disability/had/relatives/in/his family/who/wanted to/use/his organs/for/transplantation/The immediate family/had given/their/consent/but/he himself/could not/understand/the contents/of/the informed consent form/Prior to/the/course/of/study/the family's opinion/would/prevail/now/back to/consider/that/he/has turned/18/and/needs/to determine/whether/his intellectual disability/is/so severe/that/he/is unable to/exercise/his/civil rights.

Yes/For example/in the case of/intraoperative/patient/informed consent/it/used to be/thought/that/as long as/there/was/a/pre-operative/signature/if/there/was/an/unforeseen/circumstance/in/the operation/that/might/require/an/extension/of/the operation/if/there/was/a/curable/option/one/would/simply/choose/to do/that/But/now/one/would/consider/whether/this/added/operation/would/have/an impact on/the patient's quality/of/life/later on/After/the course/there/is/an/option/to/ask/the patient's family/for/further/advice.

There/should/be/There/is/an/exercise/in/thinking/for/yourself/in terms of/issues/One/does not/judge/right/and/wrong/based on/one's/subjective/experience/one/thinks about/the/theoretical/basis/such as/the application of consequentialism/etc/For example/for/AIDS/patients/many people/still/want/to/stay away/This/is/why/I/was/more/concerned about/the/protection/of/the privacy/of/AIDS/patients/

But/now/it/will/be/more/inclined to/the/real/name/management/of/AIDS/and/medical/institutions/ should/also/have/relevant records/and/pay attention to/the direction/of/the flow of/AIDS/patients.
Yes/Before/I/studied/ethics/I/might/not/necessarily/choose/the most/correct/approach/when/I/saw/ these cases/but/after/studying/I/would/at least/use/some principles/to/apply to/these cases/and/ analyse/what/is/the most/appropriate approach/from/all aspects/For example/the case/of/the hysterectomy/of/a/mentally/h/and/icapped/patient/at/the/Nantong Welfare Institution/This/was/ because/it/would/have made/the orphanage/a lot/less/of/a/problem/Before/this course/if/I/had been/ a/doctor/I/would/have agreed with/the/orphanage/and/gone to/jail/myself/But/after/I/studied/it/I/knew /that/it/was/wrong/to do so/and/to respect/her right/to/reproduce/on her own.
Of course/there/is/After/studying/ethics/I/know/that/some things/can't/be/done/or/how/to/deal with/ some situations/is/more/appropriate/For example/in a case/the director/of/a/welfare/home/asked/for/ a/hysterectomy/for/a/woman/with/IQ disorder/Would/you/like/to/have/this operation/or not This/is/ definitely/not/something/that/can/be/done/This situation/is not/the/indication/of/hysterectomy/just/for/ fear/that/she/was/sexually assaulted/by/others/and/then/an/unexpected/pregnancy/may/have caused/a/ little/reaction/so/he/wanted/to/cut off/her/uterus/across/the/board/But/it/can't/deprive/her/of/her right/ which/contains/a/physical/integrity.
I/was/deeply/impressed with/communicating with/patients/The teacher/emphasized/that/when/we/we re/explaining/the operation/plan/to/patients/the last sentence/was/to/ask/patients/Did/we/make/it/clear /instead of/asking/patients/Did/you/understand/Because/if/you/ask/the patient/if/you/understand/ anything/he/will/actually/substitute/himself/and/he/feels/that/I/don't/seem to/understand/is my fault/ and/he/may/be/even more/reluctant to/explain/what/he/didn't/understand/If/we/didn’t/make it clear/ we showed/that/we/were/more/willing to/answer questions/for/patients/There/are/also/interpersonal relationships/with/other doctors/and/nurses/There/are/also/some situations/involving/ethical issues/ that/require/your attention/and/judgment/If/there/is/any/accident/during/an operation/and/it/is not/ an urgent situation/needing rectification/we/should/send/a/doctor/from/the operating room/to/ communicate with/the patients’ relatives/asking for/their informed consent/before/deciding on/how /to continue/the surgery/I/think/this/is/still/very/important/because/it/seems/that/many/doctor-patient/ disputes/in the case/cited/by/the teacher/started/from/this part/Some doctors/will/think/that/I/am/ doing/the best/for/the patient/but/if/the/final result/is not/good/the patient/will/easily/blame/the/ doctor/Therefore/we/should/make/informed consent/in/this/respire.
I/think/you're/walking/at/an/entrance/on/the/road/You/just/know/A/is/right/and/B/is/wrong/But/now/ you/have/reason/to/tell/him/why/A/is/right/and/why/B/is/wrong/For example/the mother/of/a/ pregnant/woman/wanted/her daughter/to/abort/her child/because/she/was/worried about/the risk of/ deformity/but/the/eugenics doctor/advised/the/pregnant women/to/have a try/In other words/her family/just/didn't/want/her/to/take risks/but/professional medical/advice/thought/it/could/be/carried out/If/I/were/a/doctor/I/would/start/with/her mother/and/a/family/behind/her/Another/example/is/the case of/rescue/compatriots/Before/I/thought/it/was/a/family anyway/Induced/labor/doesn't/matter/ Later/I/realized/that/the fetus/was/also/a/person.
I/think/there/is/What/impressed/me/more/was/the class/on/the principle/of/patient/autonomy/The mother/was/a/Christian/and/was not/allowed/to/take/blood transfusion/Then/when/she/was/in/a/car accident/with/her daughter/she/prevented/the doctor/from/giving/blood transfusion/In another case/a /poor/cancer/patient/had/a/free operation/but/he/couldn't/afford/the follow-up/expenses/and/results/ Although/he/survived/he/lived/a/very/painful/life/The first case/before/class/I/may/subjectively/think/ that/I/should/refuse/the mother's request/but/I/can't/say/why/Now that/we/have/also/studied/health

law/together/this semester/I/think/it/is/better/not/to/agree with/the mother's request/from/the legal/ point of view/or/the ethical/point of view/First of all/I/think/she/is/not only/responsible/for/herself /but also/for/her daughter/If/she/decisively/gives up/her own life/because of/her faith/she/is/also/ irresponsible to/her daughter/And/the daughter/also/has/a/right/to/choose/her faith.

There/must/be/For example/in one case/when/a/believer/went/to/the hospital/for/rescue/he/would/die /if/he/did not/receive/blood transfusions/but/he/would not/accept/blood/from/other people/During /the class/The teacher/said/to respect/the patient's beliefs/if/he/really/insisted/If/I/hadn't studied/ ethics/in the first place/I'd/still/give/him/a/blood transfusion/probably/because of/the sanctity/of/life /After/studying/ethics/my views/on/a/certain matter/will/be/different/and/I/will/have/a/new perspective on/many issues/because/generally/things/have/two sides/There/was/another/case/before/It /was said/that/a/rich/man/needed/a/liver transplant/and/waited/for a long time/Then/There/was/a /brave/young/man/who/was/cut/with a knife/and/desperately/needed/a liver transplant/but/he/had/no/ money/At this time/it/is/necessary/to/carry out/a/comprehensive/consideration/from/medical factors/ and/social factors/When/a/question/like/this/is/thrown out/at once/I/really/don't know/how to choose/A/person/should/be ranked/him/and/now/there/is/another one/that/is urgently/needed/and/has/ no/money/In fact/it/is/difficult/to choose/After/studying ethics/facing/the above problems/there/will/ be/a/main/standard/and/direction/so that/I know/what/to do/and/I/will/not/be/as confused as/before.

The most impressive thing/should/be/the case/analysis/of/the group/Several/people/cooperated/to/ analyze/the case/and/then/used/some ethical viewpoints/and/their own ideas/to describe.

I/think/there/is/still/a/big/improvement/For example/in the case of/He Jiankui's gene editing/in/Chi na/before/learning/ethics/I/may/think more about/the scientist/level/and/after/learning/ethics/I/will/ analyze/it/from/the level/of/parents/and/children/Another example/is/the last question/in/the/exam/A /mother/insisted on/giving birth to/her second son/prematurely/in order to/save/her/eldest son/If/I/ hadn't studied/ethics/I/would/definitely/have to/judge/whether/the patient/had/surgical indicators/of/ premature birth/After/this course/I/would/have considered/this matter/more/from/the patient's point of view.

There/must/be/after all/after/studying/for/a/semester/I/can/have/a/basic/impression/of/some/of/the more important/basic/ethical principles/and/then/I/will/have/a more/systematic understanding/of/the future/clinical/practice process/Because/after all/there/is/no immersive experience/only/through/the form/of/cases/what/impressed/me/most/was/the group paper/that/The teacher/asked/us/to do/The topic/we/did/at that time/was/about/gene editing/and/the discussion/was/whether/we/should/edit/the genes of babies/such as/knocking out/some

AIDS-related genes/and/whether/the case/violate/some ethical principles/After/studying/ethics/I/hav e/a/better/understanding/of/those principles.

In fact/it/is/basically/to see/whether/your/three fundamental values/are/right/or/wrong/Even if/ you/have not learned/ethics/you/can/basically/judge/whether/it/is/right/or/wrong/However/after/ learning/ethics/you/will/master/these basic principles/and/treat/the problem/in/a/more systematic way/instead of/just/saying/what/ever comes to/mind/like/those/who/have/divergent thinking/ before/For example/the technology/of/surrogacy/itself/is not right/or/wrong/but/in the national conditions/of/our country/we/are/a/nation/that/pays attention to/blood ties/and/it/is/not/easy/to be accepted/among/the public/But/I/think/that/in the future/surrogacy/should/need/to be legalized/ because/it/is/a/very/real problem/and/the complete prohibition/of/surrogacy/does not/solve/this problem/but/only/avoids/this problem/If/you/blindly/avoid/it/it/is/nothing/more than/forcing/ surrogacy/into/the ground/which/may/cause/more/serious/social problems/which/is/my opinion.

I/think/my ability/to analyze with/actual cases/has improved/a lot/especially/For example/our group/has worked/on/a/project/on/the ethical issues/of/clone sheep/At that time/we/students/ made a discussion/in private/and/then/made/a/PPT/In this way/we/made/the project/easier/to understand/There/is/a lot of/ethical knowledge/and/if/the interpretation/was/all/about/boring theory/we/would not/remember/it/but/with/the teacher's examples/and/process/of/making PPT/I/ think/it/is/very conducive/to/our grasp/of/knowledge points/There/are/other courses/that/we/think/ hasty/due to/lack of time/but/there/is/plenty of time/for ethics/and/the discussion/is/sufficient.
I/have seen/dramas/similar to/the theme/of/doctors/in the past/but/after/taking/an/ethics class/you/ will/think/more/in the process/of/watching/in the reality/what/would/you/do/if/you/are/the character /does/the final character/treat patients with/the ethical requirements/I/think/should/be kept so that/ some points/will/come to mind/from/time to time/in my own life/For example/There/was/a/case/ of/a liver transplant patient/and/his wife/is/successfully matched/her mother-in-law/and/his husband's relatives/expected/his wife/to donate/but/his wife/is/actually/unwilling/At that time/the doctor/in the play/took/the other family members away/leaving/his wife/for/a/separate interview/ telling her/that/if/she/really/did not/want to/the doctor/could/help/her/cover up/and/he/could/tell/the other family members/that/his wife's liver/was not/suitable for transplantation/In fact/this/is/ similar to/a/case/in our class/The teacher/said/that/if/the patient/wanted to hide something/he/ would better/tell/his family/about/it/through/the patient/himself/However/the doctor/would not/ directly/tell/the family/whether/his wife's liver/was appropriate/and/the patient/should/have/a/high/ degree/of/autonomy.
Definitely/I/think/especially/the logical/relationship area/(great progress/has been made)/In the past/I/would/look at/things/arbitrarily/and/only/from/one side/Now/I/have improved/my logic/and/ know/how/to analyze things/This/is/a/significant/progress/I/remember/the case of/the Yulin maternity/which/I/heard about/a long time ago/when/it/just/happened/At that time/I/just/felt/that/the hospital/did not/take good care of/the mother/which/led to/a/tragic/jump/If/I/had not/studied/ ethics/I/would/have thought/it/was/the hospital's fault/But/if/I/looked at/the news/specifically/it/was/ because/the husband/of/the woman/did not/want to/have/a/caesarean section/Then/I/felt/that/maybe/ more/responsibility/was/on/the husband/because/he/caused/this/to happen/and/then/maybe/the hospital/didn't do/a good job/the hospital/didn't/take good care of/it/Now/we/will/take into account/autonomy/although/the family/can/act/as/an agent/when/the patient/himself/is/unconscious/ or/unable/to/make decisions/on his own/but/he/cannot/expand/his rights/because/he/has/this right/let /him/completely/replace/the mother's own will/to make decisions/I/think/it/is not/right/for/the hospital/at this point/the hospital/did not/exercise their rights/to/specific/intervention.
Yes/if/there/are/some ethical conflicts/now/I/will/know/how/to do/is/more reasonable/legal/For example/at the beginning/I/thought/that/I/could not/take any responsibility/if/the baby/was not/ born/But/after/studying/I/learned/that/if/the fetus/has been formed/it/cannot/be aborted/at will/I/ might/have thought/that/I/would/be inclined to/help/the mother/to/have/an abortion/according to/ her needs/and/then/I/might/consider/the weight of/the right/to/life/of/the fetus.
After/taking/the class/I/felt/that/I/knew/better/how to protect/myself/Some patients/strongly/ requested/surgery/but/it/did not/meet/the surgical indications/I/might/have thought/it/was/ok/to do it/before/I/feel/inspired/is/pretty/big/to/me/because/I/was finished/finals/just/met/an/aunt/asked/me/the problem/of/body donation/asked/me/about/my opinion/because/her mother/want to/do donation/ but/families/don't/feel good/Then/I/told/my aunt/not to worry/let/the patient/think about/it/again/do not/let/her/make a decision/early/Speak/deeper/I/could/only/see/one aspect/but/I/finished/on/the

ethics class/is/much/a/perspective-taking ability/I/do/a lot of/thinking/I/feel/that/my depth/of/ thinking/has increased/and/is/more comprehensive.
Critical reflection/may/be/more/obvious/to/me/because/I/am/about to/go to clinical/probation/and/I/ may/encounter/some situations/mentioned/in class/I/may/be suddenly alert/and/then/I/can/ communicate with/patients/in a more reasonable way/In terms of/ethical analysis/ability/I/ think/it/may/be/too professional/and/we/may not/be able to/do/it/after/a semester of/courses/ but/we/can/at least/know/some principles/and/principles/which/will/still/be improved/I/think/it's/a/ very deep one/and/I/think/I/may/have to/actually/do/it/to/find out/but/usually/I/may not/feel/it/For example/when/signing/the/informed consent/form/some/of/the patient's expressions/and/the patient's wishes/may/conflict with/our treatment plan/Before/we/might think/that/we/just/put forward/ opinions/all/in accordance with the patient's will/the patient/said/to do/so/I/do/according to/your will/can/relatively/protect/myself/But/when/I/came out of the class/I/realized/that/there/are/some situations/that/we/shouldn't do/this/that/there/are/some legal principles/that/can/be used/to protect/us /that/there's no need/to/be/so timid/that/it/actually/harms/the patient's health.
I/feel/that/I/did not/know/how/to/think about/many problems/before/but/after/the class/I/will/have/ different/views/and/ideas/according to/some basic views/and/principles/As for/the principle/of/ patient autonomy/I/might/have thought/that/the principle/of/patient autonomy/was/to/follow/the patient's own ideas/but/later/I/know/that/in China/family members' opinions/are/more often/ solicited.

Question 4 regarding ability Improvement_word split_word-frequency (connotation).txt

Patients38
Then 26
Question 19
Cases19
Ethical 12
Doctor 12
Principle 12
Surgery 10
Learn10
Class 10
Family members 9
Agree with 8
Things8
Ethics 8
Views 8
Point 8
Hospital 7
Think about 6
Teacher 6
Impression 6
Analysis 6
His wife 6
Transplantation 6

Mother 6
Judge 5
Aids 5
Willing to 5
Informed 5
Right 5
Maternal 5
Independent 5
Decided 4
Protection 4
Barriers 4
Institution 4
Gene 4
Intend to 4
For example 4
Daughter 4
Knowledge 4
Understand 4
Select 4
Process 3
Faith 3
Improve 3
Meet 3
Plan 3
After all3
Fetal 3
According to 3
Husband 3
Uterus3
Donate 3
The term 3
Blood transfusion 3
Prepare 3
Group 3
Communication 3
Removal 3
Respect 3
Law 3
Ability 3
Refused 2
Family2
Suggestion 2
Signed 2
Place 2

Serious 2
Thinking 2
Intelligence 2
Run into 2
Clear 2
Direction 2
Subjective 2
Accept 2
Discuss 2
The liver 2
Organs2
Analyze 2
Task 2
Tend to 2
Medical2
Using 2
Conflict 2
For 2
According to 2
What 2
Many2
Family 2
Theory 2
Society 2
Be badly in need of 2
Anyway, 2
Clear 2
Exercise 2
In view of the 2
System 2
The entry 2
Time 2
Depth 2
China 2
Knowledge point 2
Insist on 2
Make 2
Lead 2
Raise 2
Accident 2
Approach 2
Add 2
Risk 2
Children 2

Logic 2
Life2
Totally 2
Interpretation 2
Clinical 2
Just 2
Reality 2
Ask 3
Reasonable 2
Worry about 2
Idea 2
Just 2
Aunt 2
Comprehensive 2
Avoid 2
Level 2
Factor 2
Save 2
Master 2
Jump 1
Further 1
Report 1
Special 1
The final 1
Contact 1
Agree with 1
Topic 1
Title 1
Distribution of 1
Interview 1
Cover 1
Believers 1
Both sides of 1
Put forward1
From time to time 1
The so-called 1
At least 1
Sudden 1
Yulin 1
Contrary 1
At random1
Fellow 1
Intervention 1
Method 1

Nurse 1
Technology 1
Cesarean section 1
Dean 1
Pregnancy1
Blame 1
Express 1
Similar 1
Interpersonal 1
Not enough 1
Stay away from 1
On the road1
Take care of 1
On the basis of 1
Operation 1
Report 1
Boy 1
Criticism 1
Even 1
Underground 1
Regardless of 1
Cower 1
Early 1
Heard 1
Baby 1
Immersive 1
Divergent 1
Remember 1
In the past 1
At least 1
Instead of 1
Scientists 1
What kind of 1
Normally 1
Christians 1
Student 1
Reflect on 1
Tragedy 1
In the end of 1
Allow 1
The first son 1
Lost 1
Nantong 1
Environment 1

Advice 1
Clone 1
Reduced 1
Scope 1
Test 1
Reaction 1
Over 1
Do 1
Future 1
Deformity 1
Medical 1
Strong 1
Parents 1
Empathy 1
Press 1
Free 1
Indications 1
Ban 1
Preterm birth 1
Emphasis 1
Effect 1
Damage 1
Pain 1
Original1
In order 1
Management 1
Alert 1
Subsequent 1
Flow 1
System 1
Results 1
Relationship 1
Down 1
Health 1
Cause 1
Give priority to 1
Part 1
When it comes to 1
Pregnant woman1
Proportion 1
Application 1
In china 1
Human nature1
Position 1

Bleeding 1
Correct 1
Reason 1
Deprived 1
The interests 1
Like 1

Question 5 regarding assessment method_word split.txt
Quite/reasonable/Could be.
Available.
It/should be/largely reasonable to/suit/most/people/and I/think/it/still/fits/rather/well/But/the scores don't feel/so/transparent/people/only/know/one final mark/they/don't know/the percentage of/ each part/and they/don't know/which module/is/lacking.
It's reasonable/because/I/didn't/know about/this kind of/assessment/until/I/was/at/university/Because/ before/it/was/the final paper/that/was/the final grade/This kind of/assessment/allows/us/to study with/a purpose/and/direction/instead of/memorising/the material/at the end of the year/I/think/it/ also/tests/the students' ability/to think with/each other/communicate/and/work together/as a team.
I/think/it's/actually/quite/good/But/there is/a group paper report/in the process assessment/Because/ this/is/done by/a group/there are/always/someone who are lazy/in the process of/the group task/ or not/very/involved/in the process/but/if/other students/want to/get/high marks/they/have to/do/a little/more.
I/think/it's/OK/because/students/can/pay more attention to/their usual homework/instead of/dealing with/it/For example/if/we/write/a case study/I/think/it/will/help/us/a lot/if/it/is written down/ carefully/Only by/listening to/the video/carefully/can/we/get/a better score/in the regular quizzes.
I/think/it's/reasonable/I/think/the usual score/can/be higher/Because/the effect of/everyone/ Participating/in the discussion/will/actually/be better/not worse than/on paper/It's/fair/for/those who/do better in/class.
I/think/it's/reasonable/Because/there are/some important points/in medical ethics/that/we/need to/ remember/and then/we/can/test/them/by/examination/It should be/more about/the analytical thinking ability/which is/reflected in/the regular scores/that/accounts 40% of/the total/There is/ also/the final case presentation/I/think/it is/quite/reasonable.
For example/performance/in class/some students/may/be/more active/and/speak more/in class/so/ their scores/in this part/will be/higher/than others/But/other students/also/have/their own ideas/but /they/may not/want to/say it/under such circumstances/It is/true/that/the teacher/will/stop/for a long time/to ask/if/there are/other students/who/have/other opinions/but/no more students/stand up /and/speak/In addition/the discussion/on the platform/does not/seem to/be emphasized/by the teacher/so everyone/may/only/make up/for the end of the term/and everyone/can/also/get/this part of/the score.
I/think/it is/quite/reasonable/because/the grade/not only/depends on/the final exam/but also/on/the usual performance/Some students/will/suddenly/work hard/until/the end of the term.
This approach/fits/most/learning styles/and/is/probably/reasonable/With/marks/given for/daily activities/maybe/students/will be/more active/in the preparation/and/in-class performance/and/the pressure/on the final exam/may not/be/so great/I/think/the usual grades/can/be accounted for/a little less/and/the final paper grades/can/be accounted for/a little more/Some students/may be/

more introverted/and then/perform/less well/in class.

I/think/it is/quite reasonable/After all/learning/requires/a process/so/it/is reasonable to/have/a certain proportion of/procedural learning/After all/online self-study/still/requires/us/to have/a certain degree of/consciousness/so/it is/more important/to take/a certain percentage of/the grades/ through/different forms/not only/the final exam/but also/through/the arrangement of/some ordinary courses/I/think that/the 40% of/the grades/in the process study/will be/slightly/lower/and the final exam/will/still be/a little bit/heavy/Because/the process learning part/may not/be particularly distinct/such as/classroom performance/and/group case analysis/there will be/a little difference/Students/can/basically/get/all the grades/on the learning platform/because/everyone/will/ study/this part/more seriously.

I/think/it's/reasonable/Although/the final exam/is/certainly/very/important/but/I/think/the usual study /is/more important/In fact/we/usually/review/the basic knowledge/in many exams/and then/deal with/it/but/the thinking/and/understanding/of/this course/will be/more fruitful/in ordinary times/I/ think so.

First of all/we/learn/on the Internet/and/take/the final exam/on the Internet/now/and/I/think/we/ should/pay more attention to/our daily study/at this time/I/think/it is/a good thing/to focus on/ usual performance/which/the teacher/could/see/In fact/it seems that/every subject/this year/has increased/the weight of/usual score/In addition/Medical Ethics/pays more attention to/ordinary discussions/because/you/may/have memorized/the knowledge points/at the end of/the semester/but /you/can not/use/them/flexibly/which/is/just/a kind of/dead memorization/and can not/be applied in/practice.

I/think/it's/reasonable/After/I/went to/college/almost/every course/was evaluated/in this way/I/know /that/I/am/an introvert/and/I/will/definitely not/get/a lot of/points/in the class performance speech/ so/I/have to/bring my superiority into full play/for example/I/will/work hard/to write/the course paper/and then/be more serious/when/discussing/hoping to/make up for/this piece of score/Then/ some of the more outgoing students/dare to/speak/of course/they/will/also/bring their superiority into full play/which/are/different people/different abilities/and/personalities of/the embodiment/ all included.

I/don't think/it's/possible to/fully/reflect/it/yet/because/I/think/this proportion/is/normal/usual scores/ divided into/so many/modules/is/also/very/good/However/I/have noticed/that/there are/only/two or three/students/in/each class/who/speak/more frequently/And then/most of/the other students/only/ spoke/once or twice/or more often/not at all/However/I/think/there were/actually/many who/listen ed to/the lectures/very/carefully/but/their personalities/were shy/and did not/dare to/express/ themselves/But/not daring to/express itself/may be/a manifestation of/not being positive/enough/ But/this/also/makes/their usual scores/very/different from/those who/are/more extroverted/ in the degree of/serious study/I/think/they/are about/the same/If/I/had to/make a suggestion/I/would/ suggest/that/the percentage of/speech scores/be/slightly/smaller/but/not particularly/small.

I/think/the learning platform/maybe/not/necessarily/reflect/the learning ability/of/students/ sometimes/some people/brush it up/I/suggested/that/the platform/could/add/a little more/practice/ which/would be/more convincing.

I/think/the proportion of/assessment/is/good/that is/we/need to/interact/more/and/finish/the platform homework/especially/many students/cram/at the end of/the term/However/for/the training of/ medical ethics/which/is more inclined to/thinking/I/think/it/is better to/have/a larger proportion of /assessment/which/can/better/reflect/the actual learning process of/students.

I/think/it's/very/good/because/the final exam/will/inevitably/sometimes/have/abnormal performance/ and/it/can/be/more objective/to combine/it/normally.
Okay/Because/this course/is/mainly/about/learning/the way of thinking/sometimes/the things/in the test/may not/be/so/flexible/and/the test scores/may not/necessarily/reflect/the learning effect/It is/ better/if/it is/a case/but/some knowledge/may/be/more/rigid.

Question 5 regarding assessment method_word split_word-frequency (connotation) .txt

Classmate 16
Peacetime 16
Final 14
Grade 12
Reasonable 11
Exam 11
Learning 10
Speak 8
Process 7
Classroom 7
Seriously 6
Score 6
Platform 5
Courses 4
Cases 4
Usual 4
Group 4
Discussion 4
Assessment 4
Teacher 3
Thinking 3
Play 3
Cope with 3
Proportion 3
Pay attention to 3
Sometimes 3
Analysis 3
Recommendation 3
Ability 3
Students 2
Expression 2
Ethics 2
Effect 2
Knowledge 2
Introvert 2
Medicine 2
Extrovert 2

40% 2
Report 2
Homework 2
conform to 2
Actual 2
Flexibility 2
University 2
Modules 2
Slightly 2
Percent of 2
Times 2
Thesis 2
Dare not 2
Positive 2
Self-learning 1
Idea 1
Team 1
Part 1
Lecture 1
Method 1
Personality 1
Fair1
Careless 1
Not enough 1
Remember 1
Less than 1
Almost 1
This year 1
Material 1
Test 1
Collaboration 1
Environment 1
Paper 1
Application 1
Sufficient 1
Inspection 1
Subject 1
Combination 1
Suitable 1
Participation 1
Review 1
Make up 1
Understand 1
Arrangement 1

Training 1
Lazy 1
Communication 1
Interaction 1
Transparent 1
Show 1
Difference 1
Effort 1
Knowledge point 1
Form 1
Help 1
Rigid 1
Consciousness 1

Question 6 regarding comparing traditional classrooms_word split.txt
With/today's/teaching methods/it/is possible/to/look back at /the video/of/the knowledge points/ that/you/did not/understand/before/in a targeted manner/during/the teacher's lecture/However/in/ the pre-study stage/of/a traditional classroom/it/can/be rather general/and/most of/what/the teacher /says/in class/makes/you/feel like/the first time/to/learn/without/focus.
Course videos/can/be/played back/Traditional classes/only/have/the teacher's courseware/for/us/to/ watch/and/the knowledge content/is/not sufficiently/detailed/There/is/also/the fact/that/it/is/easier/to/ communicate/in/live class/In/a traditional classroom/you/need to/raise/your hand/and/speak/one by one/and/it/takes longer/Online live courses/are relatively free/to/speak.
Probably/more freedom/in terms of/time/The freedom/to/look back at/points of knowledge/as/you/ choose.
The class/is/actually/quite interactive/but/it/will/be/less than/the PBL class/The traditional class/is/ more inclined to/offline/whereas/the online class/emphasises/more/student's autonomy.
Because/classroom teaching/pays more attention to/this knowledge point/There/are/few/cases/ mentioned/and then/the impression/is/actually/not/so deep/It/is/mainly/that/the application/of/rich cases/in/our classroom/may/be/more prominent/In addition/there/is/mainly/such a process/of/ replaying/knowledge points/online.
Because/I/think/the form/of/live class/is/actually/very close to/our/offline mode/I/don't/feel/that/the effects of/these two learning forms/are different/However/recording and broadcasting/also/has/the advantages/some students/didn't/hear something clearly/and then/they/were/too embarrassed to/ask questions/in class/but now/they/could/go back/and/watch/it/again/In fact/all/online courses/are/ studied/and/the test questions/are/done/both first/Then/I/will/have/a systematic understanding/of/the live class/the next day/so that/I/will/know better/when/I/listen to/it/the next day/I think/what's/ good/about/the course/is/that/it's/not long/Every/video/is short/and/very suitable/to/be used as/ a preview/and/I can't/stand it/if/it's/too long.
The effect/is/similar/We/basically/have/discussions/now.
Usually/for/the sake of/continuity/of/knowledge/the teacher/will/tell/us/if/we/has/any questions/and then/ask/him/after/this part/of/his explanation/or/ask/him/after class/But/in fact/some students/such as /me/won't/go to/ask/the teacher/after class/and then/there/may/be/some questions/that/can't/be answered/Under/this mode/now/the teacher/can't/see/me/beacause/we/are/online/So/I/will/speak/and/

ask questions/boldly.
In the past/classroom teaching/had/to/be taught/on time/and/we still/have/quite/a few classes/now/ almost/every day/from morning to night/Then/if/it is/arranged/like this/now/the live class/and/ meeting class/are/arranged/in the evening/you/can/watch/the video/of/the course/at any time/the day before/In this way/the flexibility/of/the schedule/will/be higher/Personally/I think/face-to-face/ discussions/are/better than/online/and/that/can/stimulate/students' participation/and/efficiency/more I/think/that/it/is/difficult/for/students/to/absorb/it/simply/by/relying on/teachers/to/teach/During/this process/students/should/still/express/their opinions/more/such as/the ethics course/is/very good/ because/this/can/not only/deepen/the memory/but also/promote/the development/of/their own thinking.
In the past/in/the offline class/students/may/not/understand/some of/the problems/during/the process/in/the meeting class/If/it/is/an online class/after all/there/are/videos/and/records/which/are convenient/for/us/to/review/This method/is/also/more reasonable/for/some students' time/ arrangement/and/it/is/not necessary/to/study/at a specific stage/In fact/what/I am/more/looking forward to/is/that/most courses/are/offline/and then/online/accounts for/a part/Such/blended learning/may/be/more effective.
I/think/this/may/depend on/the specific course/For example/this course/focuses on/the combination /of/cases/but/some/basic medical courses/such as/anatomy/and/physiology/may/not/be well integrated with/clinical practice/Like/medical diagnostics/and/medical ethics/we/still/need/teachers/ to/explain/some/key cases/and/help/us/analyze/some/more complicated areas/This method/is/still/ relatively good.
I/think/blended teaching/can/help/us/better understand/and/remember/and/make/our impression/of/ knowledge/deeper/Like/other courses/that/are/not/integrated with/practice/it/will/take more time/to/ understand/for/us.
It/is/because of/the regular scores/we/will/be a little/more/serious/at regular learning/Otherwise/ many people/may/just/review/during the final exam.
It/is/not so easy to/find/information/on/the traditional classroom teaching/because/everyone/ answers/directly/and/ask questions/according to/his own knowledge/you/couldn't/be so quick/ while/nowadays/we/can/copy/and/paste/on the network/and/get answers/immediately/through Baidu/ Besides/some doubts/can/get feedback/in the curriculum/But/there is/one thing/that/is/not good/An online class/is/not so easy to/ask questions/after all/because/you/have to/ask one by one/and/ some questions/are remained unsolved.
Traditional classroom teaching/are/not like/this course/because/there are/videos/that/can/be based on/their own habits/such as/the speed of/note-taking/so/it/can/match/the video/which/can/be pulled /back and forth/as you needed/But/the traditional class/may/not be heard/clearly/after/the teacher/ has/finished speaking/and/sometimes/several students/have not/heard/clearly/then/this knowledge point/may/be missed/However/this year/the video teaching/mode/is/actually/more convenient/for/ introverted students/like me/to/answer questions/Normally/if/they/are/in/face to face class/they/ would/not dare to/answer questions/I/may/talk more/in class/this year.
In fact/the speech problem/I/just/mentioned/can/be improved/by/blended teaching/to/a certain extent/If/I am/in/the traditional class/I/may/not/be able to/speak/at all/because/I am/too introverted /But/I/sometimes/can/muster/courage/to/speak/in/this form/online/Because of/the online class/ everyone/is/speaking/so/you/can/speak/too/In/a traditional class/people/are/looking at/you/but/sitting/ in front of/a computer/will/be much better/Compared with/traditional classroom teaching/another

advantage/of/online teaching/is/that/videos/of/detailed knowledge points/can/be/watched repeatedly/ In addition/online teaching/allows/you/to/study/when/you/are/in/the best state/of/learning/and then/ you/can/watch/the difficult/and/important knowledge/that/you/do not/understand/repeatedly

In traditional classroom teaching/sometimes/you/can't/find/a teacher/to/ask/if/you/have/a problem/ especially/in college/If/it/is/online/there/is/usually/a group/or/live class/to/discuss with/the teacher/ so/I/think/it/will be/more helpful.

For example/cramming/at the end of/the term/or/wandering/in class/or/not easily/participating in/ the course process/our/current round/of/teaching/has/made/some improvements/to/the above problems/And/if/you/interact with/classmates/the original/curriculum groups/make/you/feel/very interesting/I/also/think/that/this/is/very good.

It/might/make/some people/who/are/not so afraid/to/speak up/because/there/is/still/a relative anonymity/online/after all/the teachers/can/only/see/your name/And then/because/you/haven't been/ to/class/you/would/feel/that/you/haven't/learned/anything/and/you/will/value/some of/these online resources/posted by/your teacher/If/it/is taught/in school/online resources/will/be less valued/ relatively/I/always/like/online teaching/because/I/can/arrange/my own time/and then/I/can/read/ some knowledge points/again and again/Sometimes/if/I/follow/the pace of/the teacher's class/the teacher/may/pass/the point/soon/but/I/may/have to/pause/and then/think/and/memorize.

Question 6 regarding comparing traditional classrooms_word split_word-frequency (connotation).txt

Teaching/Give lessons 19
Then 17
Teacher 16
Lesson 12
Classroom 12
Speech 9
Question /Doubt/Have a problem 9
Having class 8
Knowledge point 8
Time 7
Study 7
Classmates 6
Traditional 6
Live 6
Answer 6
Discussion 5
Usually 5
Quiz 5
Arrangement 5
Case 4
Process 4
Online 4
Student 4
Repeat/Again 4

Focus/Attention 4
Playback 3
Effect 3
Preview 3
Medicine 3
Knowledge 3
Combine 3
For example 3
Understand 3
Interactive 3
Meeting 3
Mode 3
Memory 3
Method 3
Free 3
Relatively 3
After all 3
Introvert/Shyly 3
Proposed /Post 3
Blended teaching/Blended 3
After class 2
In class 2
End of period 2
Benefit 2
Think 2
Record and broadcast 2
Impression 2
This Year 2
Improve 2
Traditional class 2
Form 2
Explain 2
Second 2
Stage 2
Resource 2
Ethics 2
Sometimes 2
Almost 2
Deepen 2
In the past 2
Communicate 1
Pause 1
A part 1
Entity class 1

Computer 1
Concentrate and work hard 1
Practice 1
State 1
Test question 1
Baidu 1
Clinical 1
Analyse 1
Basic lesson 1
Efficiency 1
Close to 1
Completely 1
Missed 1
Can't stand it 1
Convenient 1
Refinement 1
Basis 1
Fast 1
Seriously 1
Place 1
Difficulty 1
Thinking 1
Development 1
Method 1
Most 1
Dare not 1
Clearly 1
Face to face 1
Targeted 1
Not enough 1
Anonymous 1
Extent 1
School 1
Learned 1
Back and forth 1
Feedback 1
Convenient 1
Physiology 1
In general 1
On time 1
Exam 1
Highlight 1
Flexibility 1
Cramming 1

Excite 1

Application 1

Gaze 1

Originally 1

Pace 1

Participate in 1

Complex 1

Viewpoint 1

Most 1

Best 1

Courage 1

Mind wandering 1

Otherwise 1

Focus 1

Detailed 1

Serious 1

The day before 1

University 1

Boldly 1

System 1

Every day 1

Each 1

Export 1

Raise your hand 1

Can't find it 1

Notes 1

Match 1

According to 1

First time 1

Rich 1

Look forward to 1

Participatory 1

Promote 1

Habit 1

Be more aware of 1

Speed 1

Better 1

Help 1

Can't see it 1

Get 1

Tend to 1

Continuity 1

Any 1

Anatomy 1

A little higher 1
This round 1
Simply 1
Highlight 1
Compared to 1
Actually 1
Absorb 1
Night 1
Autonomy 1
Reasonable 1
Funny 1
Ask one by one 1
Grade 1
Choose 1
From morning to night 1
Able 1
Important point 1
Paste 1
Difference 1
Always 1
Diagnosis 1
Name 1

Question 7 regarding establishing morality education in cultivation_word split.txt

It's/OK/I/don't/think/I/have/any/deeper/insights/now/It/is/because/I/have not/yet/entered/the/clinic/In/the/future/I/may/have/a/more/dialectical/view/of/clinical/issues/which/should/guide/me/in/my/clinical/practice.

It/can/be/It/will/be/more/helpful/in/the/humanitie/spiece/It/is/reflected/in/a kind of/critical/thinking/For example/the/one/case/where/the operation/needs/to/be/extended/intraoperatively/might/be/more/considerate/of/the/patient's/rights.

The/scope/of/strengthen/moral/education/and/cultivate/people/is/relatively/broad/Ethical issues/may/only/be/a/small/module/in/the/development/of/ethics/I/was/impressed/by/the Medical Ethics Committee/and/the/doctor-patient/relationship/In the future/the/attitude/and/approach/to/patients/may be/more/comprehensive/and/the/psychological/and/spiritual/aspects/of/patients/will/also/be/considered/I/was/also/impressed/by/the/fact/that/doctors/should not/make/decisions/blindly/but/should /consider/the/consequences/communicate/with/many/people/and/if/there is/an/emergency/that/needs to be/dealt with/they/need to/catch/the/main/conflict/in a short time.

I/think/it's/OK/for/me/at least/Thinking/about/ethical/issues/from/multiple perspectives/means/that/you/are not/so/easily/swayed/by/emotions/and/then/when/you/have/this/dilemma/you/can/also/seek help/from/the Medical Ethics Committee/rather than/making/an/impulsive/decision/on/your/own

I/think/ethics/has/definitely/helped/me/improve/a lot/but/I/think/"strengthen/moral/education/and/cultivate/people"/is/too/far away/In fact/I/still/worry about/entering/the/clinical/workforce/whether/I/can/cope with/the/situation/But/now/I/know/that/I/can/request/an/opinion/from/the/hospital's/ethics/committee/immediately/whenever/I/feel/that/I/cannot/handle/the case/by/myself.

I/do/have/feeling/that/after/this/course/it/will be/very/helpful/to/me/as a whole/Apart from/clinical/work/we/actually/need to/know about/the/whole/medical/industry/and/the/medical/system.
Clinical/experiments/as well as/animal/experiments/should/conform to/ethical/requirements/which/is/the most/practical/And/some/communication/with/family members.
I/think/it's/ok/The/usual/cases/as well as/the/teacher's/usual/explanation/of/knowledge/convey/some/I/think/is/enough/Maybe/there/will be/a/better/perspective/on/things/in the future/As/the/teacher/said/we/have to/think about/problems/from/many/angles/and/aspects/When/we/may/think about/problems/in the future/or/face/some/problems/of/patients/there/will be/a/better/way/to/think/from/a/better/angle/and/then/we/can/look at/somethings/more/comprehensively/What/is/more/impressive/is/the/doctor's/special/intervention/right/For example/it/is/often/said/that/the/surgical/plan/found/during/the/operation/is not/quite/the same as/that/set/before/the/operation/and/it/is/necessary/to/let/the/patient(or/patient's/family)/know/immediately/instead of/changing/the/plan/directly/during/the/operation.
Yes/because/I/have been/suffering from/ailments/recently/I/feel/that/the/doctors/in the hospital/may be/too/fierce/If/the/full/score/is/100/I/feel/that/the/doctor/has/a/score/of/60/I/hope/they/can/achieve/a/score/of/80/I/like to/have/a/relatively/good/doctor-patient/communication/I/feel/that/I/have/too little/communication with/the/current/doctors/The/doctor/didn't/tell/me/the/side/effects/of/the/drug/and/I/went/to/the/literature/to/check/its/side/effects/Because/as/a patient/in front of/the/doctor/I/actually/feel/very/nervous/and/I/suddenly/don't/know/what/to/ask/I/think/as/a/doctor/you/should/be able to/provide/something/that/you/think/I/should/know.
At least/it/cultivates/our/spirit/of/caring/for/others/For example/it/is/more/common/for/the/family members/to/never/decide to/undergo/surgery/and/whether/the/patient/himself/wants to/undergo/surgery/I/think/it/is/possible/after/all/this/course/is/medical/ethics/and/it/teaches/students/how/to/analyze/whether/things/conform/to/moral/norms/and/whether/they/are/reasonable/more/objectively/and/comprehensively/There/is/a/documentary/called/"The Gate of Life"/Before/taking/this/course/I/was/thinking/more/from/the/patient's/side/and/felt/that/it/was not/very/optimistic/After/that/I/may be/thinking/more/about/why/the/doctor/thinks/this/way/and/whether/he/thinks/this/way/is/ethical.
I/think/it/is/definitely/possible/because/medical/ethics/itself/must/be/people-oriented/and//the teacher/has/said/it/well/in/the/general/remarks.
I/think/the/study/of/this/course/can/provide/more/important/theoretical/supportfor/the/treatment/f/some/clinical/ethical/issues/In the future/and/it/is/also/an/important/means/to/tell/us/how to/better/solve/people's/problems/clinically/What/I/have/more/experience/with/should be/the issue of/patients'/autonomy/and/doctors'/special/intervention/rights/that/is/when/patients/are/unable/to/make/their/own/decisions/should/doctors/make/decisions/on behalf of/patients/and(or)/their/families/On/this/issue/I/think/it/is/still/closely/related to/clinical/practice/But/after all/I/haven't/entered/the/clinic/may be/I/can't/feel/the same way.
I/think/it's/ok/This/course/is/to/guide/us/to/think about/some/ethical/conflicts/in/medical/treatment/Usually/we/seldom/pay attention to/this/kind of/problems/Having/this/opportunity/to/learn/this/knowledge/is/of/great/help to/the/improvement of/personal/quality/Although/you/won't/feel/much/promotion/for/a/while/its/influence on/you/will/be reflected in/all/aspects/For example/in the future/I/will/pay more attention to/communicating with/patients/and/notice/somethings/that/I/may/do/unintentionally/but/may/cause/misunderstanding/to/patients.
I/think/ethics/is/related to/health/law/The/more/legal/and/ethical/knowledge/you/have/the/more/useful/it/will be/in the future/There/are/some/realistic/scene/description/discussed/in/our/study/groups/For example/when/a/pregnant/woman/wants to/have/a/Caesarean section/but/the/family members/do not/

allow/who/should/be/the/one/that/the/doctors/listen to/And/whether/to/take/cord/blood/to/save/one's/brother's/and/on/what/basis/I/think//it/will be/useful/when/there/is/ambiguity/after/learning/it/So/I/think/it/is/very/helpful/to/us/because/it/is/practical/and/instructive.

I/may/not/have/felt/that/ethics/is/so/important/before/probably/because/I/did not/go to/the/clinic/and/after/this/course/I/found/that/it/is/actually/very/meticulous/it/penetrates/into/all/aspects/the/different/judgments/made/by/doctors/in the face of/patients/there/may be/an/element/of/ethics/but/if/a/doctor/only/rely on/their/own/moral/judgment/there/is/actually/a/certain/loophole/so/after/the/learning/of/ethics/there/is/a/deeper/understanding of/this/aspect/knowledge.

This/topic/is/too/broad/I/am not/good to/talk about/but/I/remember/when/I/was/making/a/report/at/the end of/the/conclusion/I/said//If/you/really/study/ethics/you/will/understand/that/you/have to/respect/the/rights/of/patients/including/those/principles/and/you/will not/have to/deal with/it/but/you/will/avoid/a lot of/conflicts/after/learning/them/The/truth/is/that/all of/the/university/courses/so far/related to/morality/I/find/ethics/to/be/the/most/useful/One of/the/change/brought/about/by/this/course/is/that/in the future/I/may/will/become/more/respectful of/the/rights/and/interests of/patients/in/my/clinical/contact with/patients.

I'm/sure/it/will/In fact/values/are/also/combined from/small/knowledge/points/Just like/my/view of/some/conflicts/at the beginning/it/is/different/after/learning/the/class/I/may/be/more/comfortable/with/some/disputes/in/the/clinic/or/think/more/I/often/think about/our/health/care/system/or/whether/our/medical/practices/are/in/the/interest/of/patients.

I/think/there are/For example/as/I/mentioned/before/I/feel/that/my/thinking/will not be as rigid as/before/After/taking/this/course/I/feel/that/I/will be/more/proficient/in/clinical/communication with/patients.

OK/In the future/clinical/learning/I/may/have/a/deeper/understanding/and/deal with/the/doctor-patient/relationship/with/patients/in/a/more/scientific/way/Then/I/also/know/that/there/is/such/an/organization/as/the Medical Ethics Committee/Sometimes/I/can/also/consult/and/seek help.

What/should be/important/in/medical ethics/is/the/way/in which/these/questions/are/thought about/I/think/it/will/give/me/a/deeper/understanding/of/many/aspects/of/clinical/treatment/or/doctor-patient/relationship/For example/if/a/husband/and//wife/see a doctor/and/there is/AIDS/I/have/never/thought/about/how/to/deal with/this/situation/before/but/in/the/learning/process/I/know/to/protect/the/patient's/privacy/but/to/encourage/him/to/tell/his/spouse.

Question 7 regarding establishing morality education in cultivation_word split_word-frequency (connotation).txt

Patient16

Clinic16

Problem14

Ethics12

Doctor12

Think9

Afterthat9

Ethic9

Doctor-patientrelationship6

Help6

Knowledge5

Medicaltreatment5
Communication5
Medicine5
Relationship5
Patient5
Decision4
Dealwith4
Familymember4
Ethics Committee4
Course4
Study4
Course4
Accordwith3
Strengthenmoraleducationandcultivatepeople3
Tell3
Teacher3
Perspective3
Morality/Ethics3
Profound3
Forexample3
Can/Beableto3
Practical2
Afterall2
Experiment2
Practice2
Seek2
Case2
Thinking/Mind2
Guide2
Deal with 2
Scope2
Future2
Notice2
Hospital2
In the face of 2
Plan 2
Operation/surgery2
Sideeffectc2
Atleast2
Conflict2
Exist2
Understand2
Comprehensive2
Impression2

Rights2
Contact2
Special2
Change2
Way2
Spirit2
Norm2
Judgment2
Respect2
Often2
Understanding2
Improve2
Intervention2
Allaspects2
Analyze1
Contact1
Optimistic1
Extend1
Mention1
Promotion1
Meticulous1
People-oriented1
Seeadoctor1
Combine1
Copewith1
Attitude1
Critical1
AIDS1
Caesarean1
Report1
Loophole1
Basis1
Generalremarks1
Encourage1
Dilemma1
Proficient1
Documentary1
Law1
Blindly1
Consequence1
Decision1
Pregnantwoman1
Reality1
Comprehensively1

Realm1
Benefit1
Science1
Immediately1
Quality1
Principle1
Conclusion1
Mainconflict1
Husbandandwife1
Multi-angle1
Literature1
Replace1
Protect1
System1
Right1
Discussion1
Humanity1
Avoid1
Animal1
Common1
Method1
Organization1
Autonomously1
Cultivate1
Value1
Privacy1
Topic1
Objectively1
Emotion1
Dialectical1
Impulsion1
Explain1
Convey1
Spouse1
Consult1
Umbilicalcord1
Dispute1
Reasonable1
Drug1
Support1
Solve1
Clinicaltreatment1
Nervous1
Misunderstand1

Question 8 regarding difficulties and advice_word split.txt

So far/no/it/went quite smoothly/But/I/still/think/offline courses/are better/because/the offline class room experience/is better/and/the memory points/are a bit stronger.

The course video/feels like/it will/be slightly easier/shorter/and/probably/not/covered/in enough Depth.

Some of/the live classes/are at random times/and/some are fixed/which/may/be personally demanding/in terms of/scheduling/and/some times/are more inconvenient.

Due to/the pandemic/classes/are being taught/online/Meanwhile/because of/this course/contains/ some/live classes/so/I/think/it/is possible/that/compared to/some/other classes/or/learning/will be/ more intuitive/and/then/also/we/feel/that/it/may be/more serious/In terms of/study resources/can be /in/a/clear point/At first/I/was/not sure/which book/is/the textbook/and/then/I/knew/that/the exam/ was coming up/because/I/would/normally/have taken notes/Only/later/did/I/find out/that/it/was/this book/and/I/asked/my classmates/to/find out.

Because/it/is/the suspension period/of/the epidemic/my intuitive experience/is/that/every time/I/ take/an online video class/I/take/some notes/such as/typing/a word document/There/is/no/ corresponding PPT/in/this part/so/I/feel/it's/a bit difficult/to/learn/but/in the end/it/seems/that/it's/ not/much different from/the teacher's/PPT/In addition/I/don't know/if/other students/have/read/the knowledge points/online/It/is possible/to/watch/the video lessons/while/watching/the mobile phone/ so/the learning quality/cannot/be guaranteed/Difficulty/Actually/as/I/mentioned/before/it's/the feeling/that/the key points/and/difficulties/don't/seem to be/so prominent.

If/there/is/no/live class/the course/on/Chaoxingerya/may/be/a little insufficient/for/me/Some/parts/ are /too simple/or/expansion/is/not enough/I/don't/encounter/any/difficulties/about/this course/ because/in fact/if/there/is/any/problem/I/will/definitely/reflect/and/communicate/with/the teacher/at the first time/and/the teacher's/reply/is/quite/timely/so/there/is/no problem/Basically/we/have/a/ group/for/every /course/either/WeChat group/or/QQ group/This/will/lead to/more/timely/ Communication.

I/suggest/that/teachers/use/DingTalk/would/be/more stable/and/it/can/be/played back/The Tencent meeting/used/by/the teacher/now/cannot/be/played back.

I/don't/think/there/is/any problem/I/think/the biggest problem/is/that/there/is/no need/to/repeat/small chapters/with/big/ones.

I/think/ethics/is/actual/much better/than/other classes/because/other classes/don't/necessarily/have/the form of/live classes/At/the beginning of/the class/the teacher/also/said that/this class/mainly/ teaches/us /to/think about/problems/from/an/ethical/point of view/such as/the case/given/by/the teacher/in/class/In fact/I/think/this/is/indeed/a/way/for/us/to/exercise/our/ability/to/think/from/an ethical perspective/If/there/are/deficiencies/in/the teaching/of/the course/I/mainly/want/to/talk/about/ the exam/We/don’t/know/what/to/do/when/preparing for/the exam/because/the teaching materials/ may/be/different from/the video/and/PPT/of/the teacher’s class/For example/some/chapters/have/ 6 points/and/some/have/7 points/Maybe/these/can/be/combined/and/it/seems/that/these/content tests/ will/not/be tested/It's/just/that/when/I/was/in class/I/felt/a little/confused/and/I/didn't/know/which one/to/focus on/when/preparing for/the exam.

Students/will/appear/to be/a little/more/lazy/at home/but/everything else/is/fine/The overall feeling/ is/not/comparable/to/the state of/them/in school/I/feel/that/students/may/not/revew/at home/but/at school/they/will/review/more often.

In terms of/cases/we/can/choose/more/cases/that/may/be/more controversial/in/contemporary society /Maybe/if/we/start from/these/it/may/be/more helpful/for/our/younger students/to/study/in the future.

In/live classes/sometimes/students/do not/see/the news/because/the news/was announced/the night before/and/then/not/notified/again/the next day/so that/students/forget about/it/and/miss/half an hour/of/the class/I/think/it/will/be/a bit rushed/to/have/a/live class/the next day/and/the news/was announced/the night before/There/are/no/other suggestions/I/think/this teaching model/of/medical ethics/is/very good/under/the epidemic situation.

Except/that/sometimes/the network/may/get/a little/stuck/or/something/there/is/nothing else/Some / people/prefer/the form of/online class/feeling/that/they/will/concentrate/more/Because/if/you/study/at home/there/may/be/all kinds of/distractions/and/it/is/easy/to/get distracted/It/is/suggested/that/this case-centered/way/of/explanation/can/be/maintained/in/each/class/I/think/this/is/quite good/I/think/ there/are/still/a few/people/who/interact/in class/that/answer questions/at ordinary times/Usually/ students/can/be/guided/to/answer questions/so that/everyone/can/participate/more/In fact/if/the case/ is/more interesting/or/if/the classmates/will/feel/better/about/it/they/will/want/to/share/more/Trying/to /find/some/interesting cases/or/cases/that/are/more/in line with/the current reality/but/not/the old one/which/may/be/more useful.

Students/cannot/systematically/complete/their questions/when/they/ask questions/or/they/are/easy/to be interrupted/by/students/There's/also/an issue of/self-discipline/because/some/students/just/fall asleep/during/the online class/and/wake up/to/find/the class/has/finished/while/this situation/ cannot/appear/in reality/I/think/if/it's/all/online/it's/not/as intuitive as/offline/And/when/some/ knowledge/is/in/the syllabus/but/not/in/the video/I/do not /know/whether/to learn/What's more/the teacher/didn't/highlight/the points/for/the exam/in/this course.

I/am quite accustomed/to/this model/which is/with/higher/freedom/As long as/I/am/self-conscious/ in fact/I/think/the knowledge/I/have/mastered/is/similar to/the offline class/and/I/think/there/is/no/ difference/I/think/a theory/is/three-dimensional/when/there/are/abundant/cases/to/tell/and/I/personally/ prefer/the analysis/of/cases.

Because/the teaching time/is/flexible/sometimes/you/just/get/the time/wrong/I/remember/sometimes/ it/was/morning/and/sometimes/it/was/evening/If/we/had/noticed/we/would have/avoided/it/But/ compared with/the fixed/course schedule/we/used to/have/we/would/certainly/have/the problem of/ not/remembering/the course time/because/it/is/relatively/temporary/notice.

I/don't/have/textbooks/Probably/because/I/feel/that/ethics/is/more/inclined to/the type of/thinking/it/is /not until/the middle of/the semester/to/start/the class/and/I/began/to/buy/books/at the beginning of/the semester/so/I/did not/buy/them/again/during/the study/I/watched/all/those videos/two days/ ago/because/I/enjoyed/watching/them/but/I/only/watched/them/once/there/were/no/books/to read/and/ there/was/a lack of/a/holistic/feeling/I/feel/that/the schedule of/class/is/relatively/short/so/I/think/we/ can/add/some /questions/including/explaining/some/difficult/questions/including/those/that/seem/a little/ambiguous Those/students/in/the QQ group/asked/a few/questions/those/in fact/I/am/very/ confused of/Therefore/the cases/that/may/be discussed/in/the group/or/collected/by/students/can/ also/be used/as/a basis/for/further/thinking/and/analysis/of/the course.

Online/development/depends on/students'/self-discipline/If/there/will be/both/online/class video/and/ offline class/the effect/may be/much/better than/we/all/online.

After/learning/the knowledge points/there/is/a live class/some/questions/can be/timely/answered

In/my/opinion/what/is/more difficult/is/the case/role playing/part/in/the online course/video/In fact/

I/can/hardly/hear/what/they/are/saying/clearly/The sound/in/the video/may not/be/very/good/ sometimes/it is/very/loud/sometimes/it is/very/low/which/may/affect/one of/our/learning process/In addition/I/feel/that/the textbook/is not/used/very/much/and/the content/of/the class/does not/fit/the textbook/so/I/feel/that/the textbook/is not/particularly/useful.

Question 8 regarding difficulties and advice_word split_word-frequency (connotation).txt

Question 14
Having Class 13
Case 11
Teacher 10
Then 10
Course 10
Time/Schedule 9
Online meeting Class 8
Classmates 6
Textbooks 6
Study 6
Exam 5
Ethics 5
Thinking 5
Announce 4
Students 4
Class 4
Difficulties 4
At usual 4
Not Enhough/insufficient 4
Sometimes 3
Teaching 3
Ask Questions 3
Perspectives 3
Pandemic 3
In time 3
Intuitive 3
Self-decipline 3
Answer 3
Disturb/Distruct 3
Compare 3
Chapters 3
School 2
Hard 2
Communications 2
Overall 2
After that 2
Reality 2

Such as/for example 2
Review 2
Knowledge 2
Model 2
More 2
Suggestion 2
Notes 2
Clear 2
Few 2
Effect 2
Knowledge Points 2
Form 2
Lack 2
Explain 2
Previous 2
Encounter 2
Analyse 2
Evening 2
Interesting 2
Ability 2
Remember 2
Key point 2
Play back 2
Arrange 2
Fixed 2
Contemprory 1
Process 1
All 1
Difference 1
Slightly 1
Temporary 1
Forget 1
Sleep 1
Meeting 1
Highlight 1
Concentration 1
Random 1
Self 1
Various 1
Smooth 1
At the beginning 1
That way 1
sound effect 1
Depend on 1

Chaoxingerya 1
Place 1
Seriously 1
Speech 1
During 1
Platform 1
The day before 1
freedom 1
Semester 1
Ambiguity 1
Theoretical 1
Extended 1
Controversial 1
Medical 1
Reason 1
Involvement 1
Systematic 1
Experience 1
Flexible 1
Stable 1
Avoid 1
Interactive 1
Reflect 1
Loud 1
Syllabus 1
Expand 1
Complete 1
Higher 1
Post-learning 1
In-depth 1
Center 1
No better than 1
Exercise 1
Lead 1
Enrich 1
Proper 1
Easy 1
Perception 1
Corresponding 1
Resources 1
Attention 1
Morning 1
Collect 1
Combine 1

Discuss 1
Repeat 1
Guarantee 1
Follow closely 1
Play 1
Mastery 1
Hastily 1
So far 1
